# A deep genetic structure phylogenomically frames the closest algal relatives of land plants

**DOI:** 10.1371/journal.pbio.3004005

**Published:** 2026-11-03

**Authors:** Maaike J. Bierenbroodspot, Cäcilia F. Kunz, Elisa S. Goldbecker, Maike Lorenz, Iker Irisarri, Thomas Pröschold, Tatyana Darienko, Jan de Vries

**Affiliations:** 1 Department of Applied Bioinformatics, Institute for Microbiology and Genetics, University of Göttingen, Göttingen, Germany; 2 Maastricht Centre for Systems Biology and Bioinformatics (MaCSBio), University of Maastricht, Maastricht, The Netherlands; 3 Department of Experimental Phycology and SAG Culture Collection of Algae, Albrecht-von-Haller-Institute for Plant Sciences, Universisty of Göttingen, Göttingen, Germany; 4 Department of Biodiversity and Evolutionary Biology, Museo Nacional de Ciencias Naturales (MNCN-CSIC), Madrid, Spain; 5 Research Department for Limnology, University of Innsbruck, Mondsee, Austria; 6 Campus Institute Data Science (CIDAS), University of Göttingen, Göttingen, Germany; 7 Department of Applied Bioinformatics, Göttingen Center for Molecular Biosciences (GZMB), University of Göttingen, Göttingen, Germany; University of Georgia, UNITED STATES OF AMERICA

## Abstract

Discerning the nature of the last common ancestor of land plants (embryophytes) and algae allows to retrace the adaptations that ushered in the conquest of land by plants. For this, the closest streptophyte algal relatives of embryophytes hold key information. Albeit initially coming as a surprise due to their plain body plans, all major phylogenomic efforts have concluded that the Zygnematophyceae are the algal sisters to land plants. The Zygnematophyceae are the streptophyte algal class with the greatest species richness and while we now have ample genome information on a few select members of zygnematophytes, our understanding of the genomic divergence as well as genetic potential for key traits is limited by a lack of phylodiverse data that accounts for this diversity and integrates it into a phylogenomic framework. We here sequenced 43 new transcriptome datasets for the Zygnematophyceae and built a phylogenomic tree based on a total of 104 zygnematophyceaen transcriptomes and 2243 loci. We recover a deep genetic structure for the Zygnematophyceae: Despite the deep split between Spirogyrales and their unicellular sister group Desmidiales, most Spirogyrales emerged after pronounced genetic divergence, accommodating the attainment of multicellularity and divergent traits such as unique cell and plastid division. Overall, our data capture signatures of massive ancient radiations that could only be revealed thanks to a phylodiverse sampling. Recognizing the deep genetic divergences and a vast evolutionary history of the Zygnematophyceae are key to illuminate the nature of the algal progenitors of land plants.

## Abstract 2

Understanding the algal ancestors of land plants requires resolving the deep evolutionary history of Zygnematophyceae, their closest relatives. This study shows that the group is ancient and deeply diverged, refining models of key innovations preceding plant terrestrialization.

## Introduction

As the closest algal relatives of land plants [1–6], the Zygnematophyceae have attracted considerable attention from the plant evo-devo community in recent years [7–9]. These efforts are underpinned by genome sequences of selected species [10–15]. The class Zygnematophyceae is the most species-rich of all streptophyte algae [16] and was established by Round [17] and taxonomically validated [18]. Traditionally, the class was divided into two orders, the filamentous Zygnematales and the unicellular Desmidiales, based on the morphology of vegetative cells [18,19]. Recently, based on a phylogenomic tree of 326 loci, Zygnematophyceae were divided into a five-order system [16] (Desmidiales, Spirogyrales, Zygnematales, Serritaeniales, and Spirogloeales). While each order was well-supported, the phylogenetic relationship among them remained open and orders were in some cases represented by low genetic diversity and/or limited taxon sampling. To address this, we used a systematic sampling approach, aiming for covering increased phylodiversity in Zygnematophyceae across taxonomic groups, growth types, habitats, and culture collections.

We obtained 43 strains from which we generated de novo transcriptomes. Combined with publicly available data, we assembled a dataset of 104 zygnematophyceaen algae, nine other streptophyte algae, eight land plants, and four chlorophyte algae as outgroup. Building on our previously established methodological approaches [20,21] and using IQ-Tree v3 we computed a phylogenomic tree based on 2243 loci and a site-heterogeneous best-fit model [22] with Posterior Mean Site Frequency (PMSF) [23] profiling (LG + C60 + F + G-PMSF) (Fig 1).

**Fig 1:**
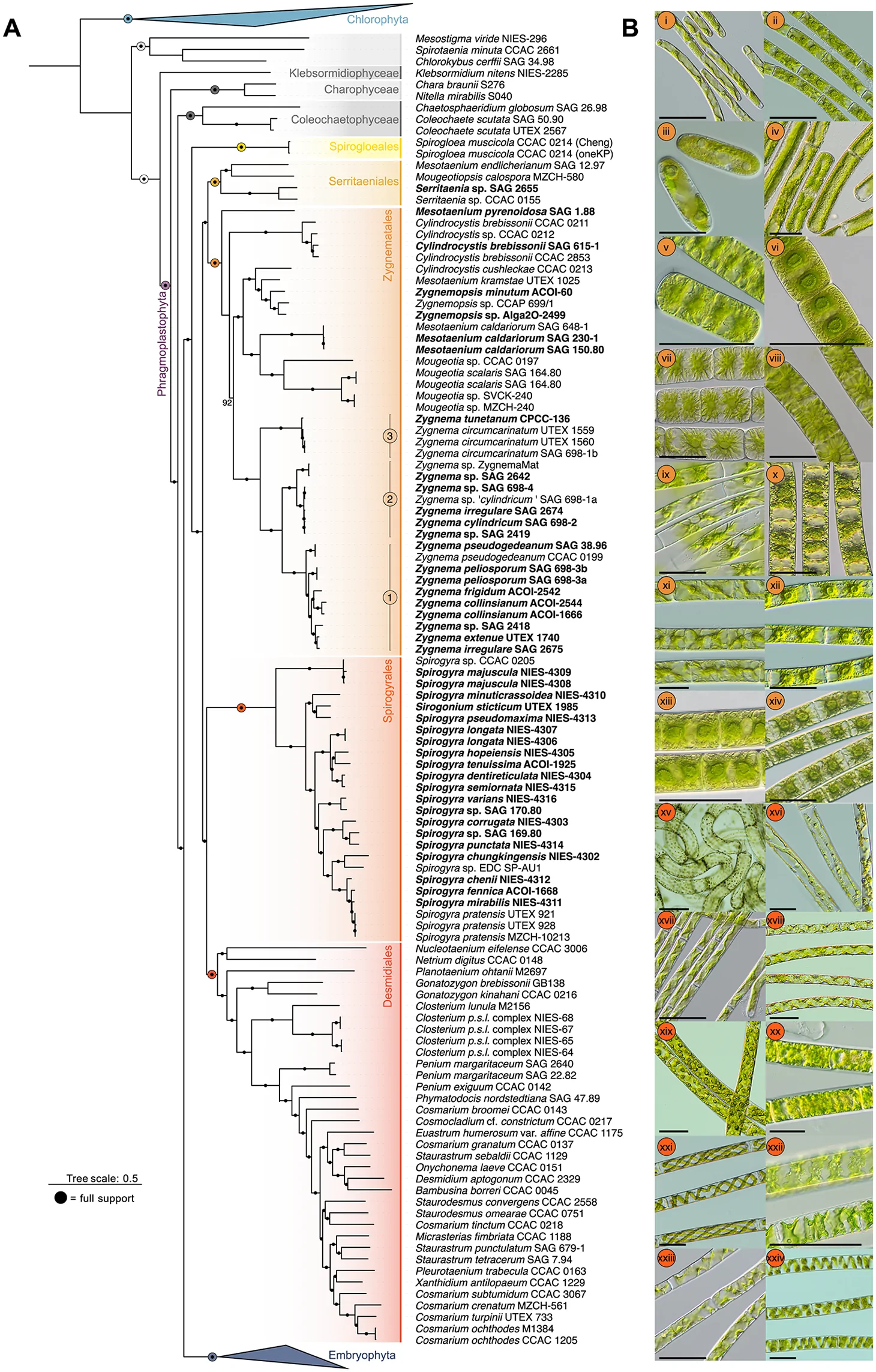
A robust phylogenomic framework and deep genetic structure within Zygnematophyceae. **(A)** A maximum likelihood phylogenomic tree based on 2243 loci. The Zygnematophyceae are divided into five orders: **(i)** Spirogloeales, **(ii)** Serritaeniales, **(iii)** Zygnematales, **(iv)** Spirogyrales, and **(v)** Desmidiales. Filled dots mark full support and the colors correspond to the labels of major monophyla. **(B)** Light micrographs showcasing the newly sequenced diversity: **(i)** *Mesotaenium kramstae* NIES-657; **(ii)** *Zygnemopsis minutum* ACOI-60; **(iii)** *Mesotaenium caldariorum* SAG 230-1; **(iv)** *Mougeotia scalaris* SAG 164.80; **(v)** *Zygnema circumcarinatum* SAG 698-1b; **(vi)** *Zygnema* sp. SAG 698-4; **(vii)** *Zygnema cf. cylindricum* SAG 698-1a; **(viii)** *Zygnema cylindricum* SAG 698-2; **(ix)** *Zygnema pseudogedeanum* SAG 38.96; **(x)** *Zygnema peliosporum* SAG 698-3a; **(xi)** *Zygnema frigidum* ACOI-2542; **(xii)** *Zygnema colinsianum* ACOI-1666; **(xiii)** *Zygnema* sp. SAG 2418; **(xiv)** *Zygnema* sp. SAG 2675; **(xv)** *Spirogyra majuscula* NIES-4308; **(xvi)** *Sirogonium sticticum* UTEX 1985; **(xvii)** *Spirogyra tenuissima* ACOI-1925; **(xviii)** *Spirogyra dentireticulata* NIES-4304; **(xix)** *Spirogyra corrugata* NIES-4303; **(xx)** *Spirogyra* sp*.* SAG 170.80; **(xxi)** *Spirogyra chunkingensis* NIES-4302; **(xxii)** *Spirogyra* sp*.* SAG 169.80; **(xxiii)** *Spirogyra fennica* ACOI-1668; **(xxiv)** *Spirogyra pratensis* MZCH-10213. All scalebars except for those in panels iii and xv are 50 µm. Scalebar iii represents 20 µm and the one in xv represents 200 µm. The data and code needed to generate this Figure can be found in https://zenodo.org/records/18392035 as well as https://doi.org/10.5281/zenodo.22172294 on Zenodo. Bold font highlights the new transcriptomes generated in this study.

### The phylogenomic framework of Zygnematophyceae is robust

Our tree recovered the well-accepted gross topology of the Chloroplastida, with deeply split monophyletic chlorophytes and streptophytes, six monophyletic streptophyte algal classes, and the zygnematophytes as sister to land plants [1–5] (Fig 1). Our sampling effort focused on increasing the representation especially for the orders Spirogyrales and Zygnematales to improve the phylogenomic resolution of our analyses.

The traditional order Zygnematales was split into three orders as in ref [16]: Spirogyrales, Serritaeniales, and Zygnematales (in revised form), which included the transfer of unicellular taxa (*Mesotaenium* Naegeli, *Cylindrocystis* Meneghini ex De Bary) to these new orders. Most of the other unicellular taxa remained in the Desmidiales. The cells of these Desmidiales, characteristic of a desmid cell, are typically unicellular and composed of two symmetrical semicells joined at a central isthmus. Each semicell generally contains a chloroplast, and the cells exhibit diverse shapes, ranging from simple elongate or spherical forms to highly ornamented, lobed, or deeply constricted morphologies.

Interestingly, the fifth order Spirogloeales, remains represented by a single species, *Spirogloea muscicola* (De Bary) Melkonian, which was originally assigned as *Spirotaenia bryophila* (Brébisson) Lütkemüller [10]. Overall, our phylogenomic study confirmed the five-order system [16], but Zygnematales and Serritaeniales were recovered as sister groups in our analyses based on increased sampling of taxa and genes. Additional sampling of taxa such as members of the genera *Ancylonema* Berggren*, Zygogonium* Kützing [24]*,* and organisms that resemble *Mesotaenium* (which likely is a polyphyletic genus), closely related based on preliminary *rbc*L phylogeny and genomic signatures [25–27], could further change the topology of the orders Zygnematales and Serritaeniales and/or modify the orders as currently defined.

Using the phylogenomic tree as a backbone, we performed ancestral character state reconstruction (ACSR) to infer the evolution of growth types across Zygnematophyceae (Fig 2). For ACSR, we considered the morphological capabilities of all organisms when coding the characters, although these characters primarily reflect the capabilities of the vegetative cells. In Zygnematophyceae in particular, the life cycle does not influence their overall growth capabilities, as it comprises only vegetative cells and zygospores. Based on the current sampling, of the seven transitions to filamentous growth, only two are ancient: in the last common ancestor (LCA) of Spirogyrales and the genus *Zygnema*—to both of which we turn in the following.

**Fig 2:**
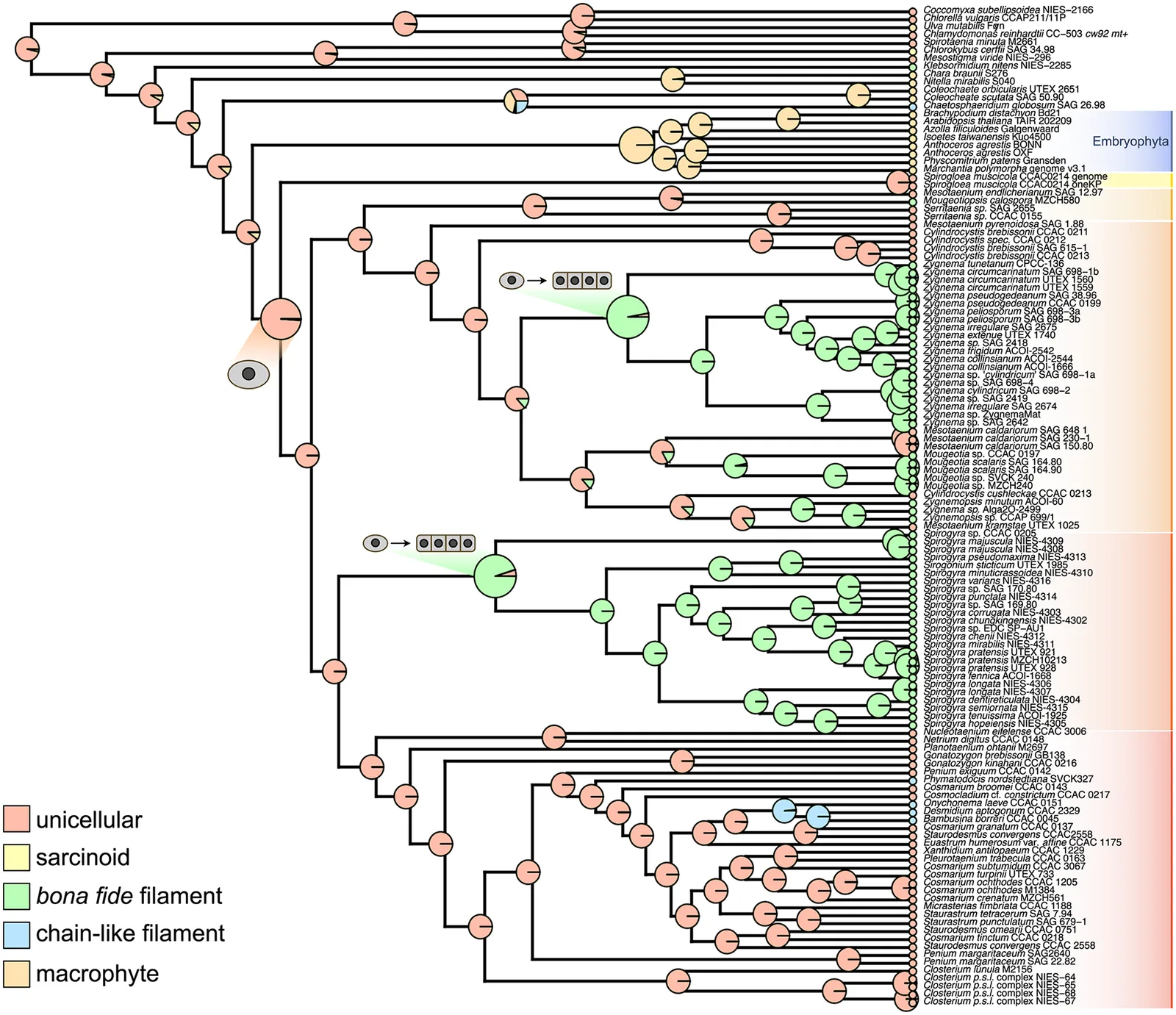
Ancestral character state reconstruction of body plan evolution across Zygnematophyceae. Growth types were coded as five-character state distributions to trace the evolution of different homologies of growth types: salmon = unicellular, yellow = sarcinoid, green = *bona fide* filamentous growth, blue = chain-like filaments (desmids), and orange = complex multicellular macrophytes. The pie charts present the posterior probabilities (PP) for an inferred growth type at a node. The inferred ancestral unicellular state for the LCA of all Zygnematophyceae (PP of 0.988) is highlighted as well as the two transitions to filamentous growth types that lead to the LCAs of the genus *Zygnema* (PP of 0.970) and the order Spirogyrales (PP of 0.950). Note that the focus here was on the Zygnematophyceae character states and results on any other lineage should be interpreted with caution. Furthermore, note that a PP of ≥ 0.95 is generally considered as the standard threshold for a robust or strongly supported ancestral state at a given node and values ≥0.99 represent very high or decisive support. The data and code needed to generate this Figure can be found in https://zenodo.org/records/18392035 as well as https://doi.org/10.5281/zenodo.22172294 on Zenodo.

### Pronounced genetic divergence before the emergence of extant Spirogyrales

The order Spirogyrales currently consists of the single genus *Spirogyra* Link. Its most iconic feature is its spiral chloroplast(s), undulating through the entire length of the cell. From the almost 400 species described, the genome of one species (*Spirogyra pratensis* Transeau) has been sequenced [15]. This single genome [15]—bolstered by the confirmation of patterns from previous draft sequencing data [28]—uncovered conspicuous deviations from other zygnematophytes, evident by genomic streamlining and pronounced genetic divergence described in Goldbecker and colleagues [15]; the most palpable example being the absence of the plastid division machinery otherwise conserved across Archaeplastida [15]. We thus used our phylogenomic framework to contextualize this divergence.

The only reliable way of distinguishing the different *Spirogyra* species based on morphology is by observing the formation of zygotes (zygospore) and their characteristics [29]. While the induction of the sexual reproduction has not been reported on all strains—and thus *Spirogyra* strains are unidentified at the species level—reducing nutrient availability has succeeded in many cases [30,31]. Takano and colleagues [30] identified 13 species based on experiments inducing sexual reproduction. They sequenced two plastid-coding markers (*rbc*L and *atp*B) and discovered seven clades (I-VII). Our phylogenomic tree recovered seven highly supported clades of *Spirogyra* spp., which correspond to those established by Takano and colleagues [30] with one exception: clade VI is missing because no strain of this clade is available in public culture collections. A subdivision into eight clades was found using SSU rDNA sequences (clades A-H; Chen and colleagues [32]). Unfortunately, no strain of this study is available and almost none was assigned to a species level because of the lack of zygospores in culture. Both of these studies confirmed that the genus *Sirogonium* Kützing 1843 belonged to *Spirogyra* (clade II in Takano and colleagues [30] or clade C in Chen and colleagues [32]). This genus differed from *Spirogyra* by 2–10 ribbon-like chloroplasts, a feature that derives from spiral chloroplast(s) based on phylogenetic and phylogenomic studies. In addition, one new clade was uncovered by our phylogenomic study containing three strains (NIES-4303 *Sp. corrugata* Transeau, NIES-4314 *Sp. punctata* Cleve and SAG 169.80 *Spirogyra* sp.). Both NIES strains were not assigned to any clade in the study by Takano and colleagues [30] (2019). In the future, our phylogenomic backbone can be integrated with phylodiverse and detailed analyses of zygospore morphology, cell wall chemistry, and three-dimensional architecture [33]; this will allow resolving the functional significance, evolutionary variability, and adaptive roles of zygnematophycean zygospore walls, an area of research that remains in its infancy despite recent methodological advances and emerging evidence for previously unrecognized cell wall complexity [34].

Overall, our phylogenomic tree included a phylodiversity of 25 different *Spirogyra* taxa. They form a monophylum, the order Spirogyrales, that branches off from the sister order Desmidiales. The branch leading to extant Spirogyrales is remarkably long (6.2 times longer than the branch leading to Desmidiales). This pronounced branch length may reflect several, non-mutually exclusive evolutionary and phylogenetic scenarios, including substantial extinction within Spirogyrales, the existence of unsampled and potentially undescribed diversity, or accelerated rates of molecular evolution along the lineage. Long branches may also arise from heterogeneous selection pressures, compositional biases, or biases associated with the use of transcriptome-derived data. Thus, the current phylogenetic pattern does not by itself allow us to distinguish among these alternative explanations. If the long branch primarily reflects extinction and/or unsampled diversity, it would imply that substantial evolutionary history is missing between the common ancestor of extant Spirogyrales and its living representatives. Under this scenario, the emergence of characteristic Spirogyrales traits—including spiral chloroplasts, genomic streamlining, and distinctive cell-division machineries—may have occurred prior to the LCA of extant Spirogyrales and subsequently been retained across the surviving lineages. This interpretation is consistent with the ACSR, which reconstructs ancient filamentous growth forms within Spirogyrales (Fig 2). However, given the alternative explanations for the observed long branch, these trait-evolutionary inferences should be regarded as hypotheses that will require broader taxon sampling and improved genomic resources to test.

### Deep genetic structure and recent bursts of radiations in the genus *Zygnema*

The genus *Zygnema* C. Agardh is characterized by two asteroid-shaped chloroplasts, each with a single central pyrenoid; we recover them as ancestrally filamentous (Fig 2). One hundred thirty-seven species are described based on features of zygospores [29]. Phylogenetic analyses of *rbc*L sequences revealed that all thus far investigated specimens were distributed into three clades [35,36]. Our phylogenomic analyses confirmed this subdivision. Three groups were pinpointed: *Z. circumcarinatum*-, *Z. cylindricum*- and *Z. peliosporum*-groups. The first clade contains only two species *Z. circumcarinatum* Czurda (SAG 698-1b) and *Z. tunetanum* (Gauthier-Lièvre) Stancheva, J.D. Hall, McCourt and Sheath (CPCC-136) the latter originally assigned to the genus *Zygogonium*. The second clade consists of several species, some not assigned at the species level. Interestingly, the strain SAG 698-1a, originally assigned as the “b” mating type of *Z. circumcarinatum* (now *Z.* sp. ‘*cylindricum*’) belongs to this clade, which indicated a past mistransfer in algal collections [12]. Most of the investigated strains are members of the third clade.

Linking molecular data with zygospore characteristics remains challenging in the Zygnematophyceae. Although mesosporium color has been proposed as a diagnostic trait [35], exceptions exist and color variation may partly reflect zygospore maturation. To date, detailed ultrastructural characterization of zygospores combined with molecular identification, including Focused Ion Beam Scanning Electron Microscope (FIB-SEM) reconstruction, has been reported for only a limited number of taxa [33,37]. For example, *Zygogonium vaginatum* SAG 2418 [36] and *Z. peliosporum* cluster within subgroup 1 (Fig 1), an attractive pair where unequivocal molecular identification can be integrated with morphological, ultrastructural, and biochemical analyses of zygospores. Publicly available strains with abundant zygospore production, such as the Svalbard *Zygnemopsis* material described by Pichrtová and colleagues [38], represent valuable resources for future studies, whereas access to some divergent filamentous genera (e.g., *Temnogametum* [39]) remains more limited.

The sister group of Zygnematales in our phylogenomic study is the order Serritaeniales [16]. This order includes (i) several taxa assigned to the genus *Serritaenia* A.Busch and S.Hess [25], (ii) the type species of the genus *Mesotaenium*, *M. endlicherianum* Naegeli, and (iii) *Mougeotiopsis calospora* Palla. Interestingly, *Mougeotiopsis* represents the only filamentous taxon within this order so far. The other specimens of this order have unicellular *Mesotaenium*-like morphologies. The genus *Mesotaenium* is polyphyletic as currently described. For example, *M. pyrenoidosum* (P.A.Broady) Petlovany and *M. kramstae* Lemmermann belong to different lineages of the Zygnematales. The diversity of *Mesotaenium*-like organisms is much higher than expected and awaits taxonomic revisions [25].

Our phylogenomic data recovers the genus *Zygnema* as monophyletic. In previous *rbc*L phylogenies [25], *Zygnema* was placed in an unsupported larger group consisting of other species of *Cylindrocystis*, different lineages of *Mesotaenium* (Meso-9 and Meso-10), and *Zygnemopsis* (Skuja) Transeau. Our phylogenomic study now robustly resolves their relationships; three additional, well-supported lineages beside the *Zygnema* subclade are recovered: (i) *Zygnemopsis* together with *Cylindrocystis cushleckae* A.J. Brook and *Mesotaenium kramstae*, (ii) *Mougeotia* C. Agardh and *Mesotaenium caldariorum* (Lagerheim) Hansgirg (Meso-9), (iii) *Cylindrocystis brebissonii*, (iv) and *Mesotaenium pyrenoidosum* (Meso-4) sister to all other Zygnematales. Both genera *Zygnema* and *Zygnemopsis* cannot be discerned by looking at the vegetative filaments, but by the presence of mucilage surrounding the gametangia in *Zygnemopsis*. Compared to *Zygnema* and *Spirogyra*, *Zygnemopsis* is a small genus consisting of 42 species [29]. In our analyses, the investigated strains are separated from *Zygnema* confirming that this morphological feature seems apt for differentiation of both genera if sexual stages are present. Unfortunately, no sexual reproduction was observed for the isolates included in the tree and therefore, no species assignment was possible.

Based on the topology of our phylogenomic tree, we made two observations for the extant diversity represented by the genus *Zygnema*. First, the clades within the genus are deeply divergent from one another—aligning with the molecular clock analysis of Feng and colleagues [12] that dated the divergence between *Zygnema circumcarinatum* and *Zygnema cylindricum* Transeau (see also [40]) to have occurred 236 ± 5 million years ago. Second, each clade experienced pronounced radiation, giving rise to the diversity captured by our dataset.

### Order-specific occurrence of signature genes shared by land plants and zygnematophytes

Traditionally, other streptophyte algal lineages than Zygnematophyceae were considered as closest algal relatives of land plants. This was owed to single-gene phylogenies and the complexity of body plans found in genera like *Chara* L. and *Coleochaete* Brébisson; yet, both in the case of Charophyceae and the discoidal *Coloechaete* species, the complexity in body plans are likely the result of secondary gains [21,41]; for a recent review on the multiple evolutionary emergences of (sometimes very complex) multicellular streptophyte bodies, see Darienko and colleagues [42]. The genomic revolution has not only phylogenomically established the sister group relationship of Zygnematophyceae and embryophytes [1–3,43] but comparative genomics pinpointed to several genetic synapomorphies in their ancestor [44].

Abscisic acid (ABA) is the foremost phytohormone in stress signaling, mounting a myriad of responses that adjust the biology of land plants to terrestrial challenges [45]. The genes homologous to those coding for the canonical ABA receptor [46–52] PYRABACTIN RESISTANCE1/PYR1-LIKE/REGULATORY COMPONENTS OF ABA RECEPTOR (PYR/PYL/RCAR). Homologous genes of *PYR/PYL/RCAR* were once considered to occur only among land plants; yet, with the advent of transcriptome [44] and genome [10,12] sequencing of zygnematophytes *PYR/PYL/RCAR* genes were found in these closest algal relatives of land plants and thus emerged as a potential molecular synapomorphy defining the clade of zygnematophytes and land plants. Among zygnematophytes, they are found in the genomes of *Mesotaenium* [10] and *Zygnema* [12,44] but not in that of the desmids *Penium* Brébisson ex Ralfs [11] and *Closterium* Nitzsch ex Ralfs [13]. Considering this patchy recovery of signature genes shared by land plants and zygnematophytes, coding capacity in genome space of the Zygnematophyceae has been insufficiently covered only by one or two genomes per order. We here made use of our largely extended sampling to explore the occurrence of *PYR/PYL/RCAR* homologs in zygnematophytes. Our maximum likelihood phylogeny recovers that the characterized [53,54] *PYL* homologs of *Mesotaenium endlicherianum* SAG 12.97 and *Zygnema circumcarinatum* SAG 698-1b fall into monophyla of *PYL* homologs of Serritaeniales and Zygnematales, respectively (Fig 3A)—including homologs in, e.g., *Zygnemopsis*, which is within the order Zygnematales and distantly related to the model strains of *Zygnema*. Yet, despite our vastly extended dataset, we did not detect homologs in any other order, suggesting potential order-specific losses of *PYL* (one in Spirogloeales and one in the common ancestor of Desmidiales and Spirogyrales); confirming this absence will require broader taxonomic sampling, supported by contiguous genome assemblies rather than relying largely on transcriptomic data. That being said, the lineage-specific occurrence of *PYL* in zygnematophytes aligns with the idea that extensive rewiring in the homologous chassis of PYL–PP2C–SnRK2 occurred—and that this likely does not constitute the core cascade [55,56]. This is further in line with our view on the modular assembly of other phytohormone signaling systems as in the case of auxin [57–63].

**Fig 3:**
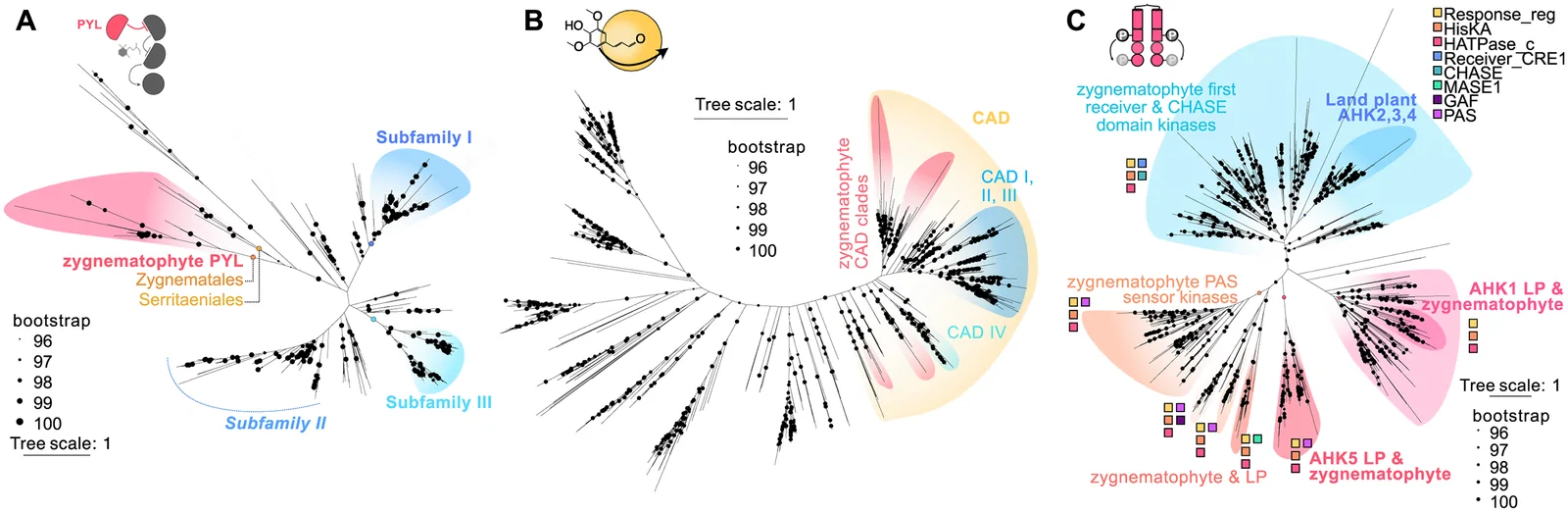
Order-specific radiations in land plant heritage genes. Maximum likelihood phylogenies of protein-coding genes homologous to signature regulators of the molecular biology of land plants. **(A)** Phylogeny of *PYRABACTIN RESISTANCE1/PYR1-LIKE/REGULATORY COMPONENTS OF ABA RECEPTOR* (*PYR/PYL/RCAR*) homologs, radiating independently of the land plants subfamilies (cf. Park and colleagues [50] and Zimran and colleagues [54]) in Zygnematophyceae and limited to the orders Serritaeniales and Zygnematales. **(B)** Phylogeny of *CINNAMYL ALCOHOL DEHYDROGENASE* (*CAD*), showing several order-specific clades and radiations; homologs were recovered across orders. **(C)** Phylogeny of histidine kinase homologs and their domain structure. PFAM domains were annotated using interproscan and consolidated per clade. PF00072: Response regulator receiver domain (Response_reg), PF00512: His Kinase A (phosphor-acceptor) domain (HisKA), PF02518: Histidine kinase-, DNA gyrase B-, and HSP90-like ATPase (HATPase_c), PF03924: CHASE domain, PF24896: AHK4/CRE1/WOL first receiver domain (Receiver_CRE1), PF05231: MASE1, PF13185: GAF domain, PF00989: PAS fold (PAS), PF08447: PAS fold (PAS_3), PF08448: PAS fold (PAS_4), PF13188: PAS domain (PAS_8), PF13426: PAS domain (PAS_9). For the fully labeled trees, see S1 Fig. The data and code needed to generate this Figure can be found in https://zenodo.org/records/18392035 as well as https://doi.org/10.5281/zenodo.22172294 on Zenodo.

Another set of land plant heritage genes concerns the chassis of the phenylpropanoid pathway. The phenylpropanoid pathway is key to a myriad of aspects of embryophyte biology, leading to the production of compounds crucial for biotic and abiotic stress response [64,65] as well as structural compounds such as lignin [66,67]. Tracing its evolutionary origin to a chassis that emerged in algae is thus a long-standing question in the field [68–73]. Streptophyte algae not only have genes homologous to those coding for a series of enzymes that have assembled in land plants into the phenylpropanoid pathway, but in some cases these homologs have even a radiated repertoire [74]. *CINNAMYL ALCOHOL DEHYDROGENASE* (*CAD*) is a point in case [74]. CAD is the last step in monolignol biosynthesis, reducing diverse cinnamyl aldehydes into their corresponding cinnamyl alcohols [75,76]. Divergent homologs of *CAD* occur even in chlorophytes but underwent massive radiation in streptophytes [74].

Homologs of this NADP(H)-dependent enzyme have been classified into three to five different groups or classes. Generally, *bona fide* [77] or class I CADs show a clear response to stress [78–81] with the highest catalytic activity [78,82,83] and with the full catalytic motif set [84,85]. The other groups or classes describe CAD-like isoforms with generally a lower (Class II) [78,82,83,86] to an even lower or no catalytic activity (Class III) [82,86] or other functions. We here use the classification from Saballos and colleagues [87], where group IV was described as monocot-specific.

None of the *CAD* homologs of Zygnematophyceae fall within the embryophyte classes, but instead form several monophyla, each containing a rich diversity (Fig 3B). This bolsters the notion that CAD was present in the LCA of zygnematophytes and land plants [73,74,88]. But as the function and structures of CAD homologs vary between the classes, it remains to be experimentally determined which substrates the zygnematophyte CAD homologs accept and under which kinetics they catalyze them. Protein structure predictions hint that streptophyte algal CADs are more like sinapyl alcohol dehydrogenases (SADs) [70]. However, given that (a) *CAD* homologs are differentially regulated in diverse zygnematophytes exposed to stressors [89,90] and (b) radiated in zygnematophytes, CAD seems to be a potential key player contributing to the chemodiversity of zygnematophytes.

Phosphorelay-based signaling is key in molecular physiological adjustments of land plants. In this response system, histidine kinases homologous to the ARABIDOPSIS HISTIDINE KINASES (AHK) are major players, acting in cytokinin-based signaling [91–93], their connections to stress response [94], and frequent responders in transcriptome data of stressed zygnematophytes [90,95–97]. Histidine kinases are ancient signaling proteins that originated in bacteria as part of two-component systems, which typically consist of a sensor histidine kinase and a response regulator. The diversity of histidine kinases expanded in streptophytes, including streptophyte algae; for example, analyses of the *Klebsormidium nitens* genome revealed a surprisingly large repertoire of HKs (e.g., ~67 in *K. nitens*) [98]. While histidine kinase family underwent significant radiation and functional specialization, marked by the emergence of distinct subfamilies with specialized roles such as cytokinin perception (CHKs), ethylene and ABA Signaling (ETR-HKs), many HK groups critical and potentially unique to land plants, such as AHK1, CKR, and ETR, have homologs in streptophyte algae.

Our maximum likelihood phylogenetic analysis recovers massive expansions in zygnematophyte AHKs in a lineage-dependent manner (Fig 3C). All clades in the gene tree contain proteins with Response regulator receiver domains (Response_reg), His Kinase A (phosphor-acceptor) domains (HisKA) and Histidine kinase-, DNA gyrase B-, and HSP90-like ATPase Histidine Kinase domains (HATPase_c). Yet, we also recover AHK1 and AHK5 as clear orthologous monophyla, whilst there are several co-orthologs to AHK2,3,4. In *Arabidopsis*, AHK2, 3 and 4 bind cytokinin via their extracellular CHASE domain [92,99,100]. Next to the land plant-specific clade there are large expansions in zygnematophyte histidine kinases containing CHASE domains. We identified several zygnemtatophyte histidine kinases that contain a PAS fold or domain. These domains are widespread across the tree of life and play a role in light sensing in plants [101]. PAS domain-containing histidine kinases that are not phytochromes also exist in *Physcomitrium patens*, other non-seed plants as well as in *Klebsormidium nitens*; in *Physcomitrium* they are regulated by light and play a role in gametophore development [102]. We recovered three of the five *Klebsormidum* PAS-kinases identified by Ryo and colleagues [102] in our dataset within the zygnematophyte PAS kinase clade. Our analysis shows that PAS-domain-containing histidine kinases are abundant and likely to be important in zygnematophytes. We also identified histidine kinases with PAS domains in the clade containing AHK5. The majority of the PAS domains correspond to the zygnematophyte sequences within that clade. On balance, Zygnematophyceae have a rich and diversified repertoire of AHKs, likely speaking of their extensive intracellular signaling.

Overall, our data capture the shared occurrence of what has been coined [41] ‘land plant heritage genes’—genes that constitute a toolkit key for signature traits of land plants. However, this shared molecular toolkit is, considering loss and divergence, only graspable by using a phylodiverse dataset [14]. With our vastly extended sampling of functional genomic space, several order-dependent patterns of loss and expansion can be gleaned—simultaneously bolstering the need for further whole-genome sequencing efforts into zygnematophyte diversity.

### Concluding remarks: Dynamic trait evolution in zygnematophytes

We inferred a robust phylogenomic framework for zygnematophytes, the closest algal relatives of land plants that pays homage to their species richness. While this represents a comprehensive dataset, it still captures a fraction of the diversity of more than 4,000 species, including hyperdiverse Desmidiales and unculturable extremophilic zygnematophytes [103]. That said, our framework recovers pronounced deep genetic divergences within Zygnematophyceae. This is not only evident from the genetic distances in the tree, but through the order-specific occurrence and within-order radiations, of genes for signature traits, and strong reductive evolutionary forces acting on the genomes [14]. This is mirrored by the diversity in cellular features and growth patterns, including repeated shifts between uni- and multicellular body plans [12,16], division patterns [104], plastid morphology [15], and more. Our data bolster the concept that the LCA of Zygnematophyceae was an ancestral unicell (Fig 2), which our analyses recover with a posterior probability of 0.988 (the body plan of the LCA of land plants and Zygnematophyceae could not be robustly inferred). Transitions to multicellularity occurred several times independently, with a few ancient transitions in the LCAs of classical filamentous genera such as *Spirogyra*, *Zygnema*, and *Mougeotia* (Fig 2). Such multiple transitions to multicellularity suggest that they are underpinned by modes of rapid evolution—conceivably through the rewiring of existing components, facilitated by changes in regulation of gene expression and protein–protein interaction network [42]. This is further complicated by zygnematophytes not only including *bona fide* filaments but also the chain-like filaments of desmid cells that are brought about by diverse and complex modes of division [105]; such chain-like filaments of desmid cells consist of individual, typically unicellular desmid cells arranged end-to-end, forming elongated, unbranched chains. Adjacent cells remain connected following cell division, while retaining their characteristic desmid morphology, often with distinct semicells separated by a median constriction. The homology of the cytokinetic mechanisms that bring about this diverse cell biology remains obscure.

Overall, we infer seven transitions to filamentous body plans, to which broader sampling of diversity will likely add more. A comparative and complementing phylodiverse approach that accounts for loss and lability [14] (some of which might be environmentally inducible [106]) is thus needed to reliably define synapomorphies of the LCA of algae and all land plants.

## Methods

### Resource table

**Table:** 

REAGENT or RESOURCE	SOURCE	IDENTIFIER
Critical Commercial Assays		
DNAse I	Thermo Fisher, Waltham, MA, USA	N/A
Sigma’s Spectrum Plant Total RNA Kit	Sigma, Saint Louis, MO, USA	N/A
Deposited Data		
Amborella trichopoda *only for gene family analysis	Amborella Genome Project [107]	https://www.ncbi.nlm.nih.gov/bioproject/212863
*Anthoceros agrestis* BONN	Li and colleagues [108]	https://www.hornworts.uzh.ch/static/jbrowse/?data=a_agr_bonn
*Anthoceros agrestis* OXF	Li and colleagues [108]	https://www.hornworts.uzh.ch/static/jbrowse/?data=a_agr_oxford
Anthoceros punctatus *only for gene family analysis	Li and colleagues [108]	https://www.hornworts.uzh.ch/en.html
*Arabidopsis thaliana* genome TAIR V10	TAIR [109]	http://www.arabidopsis.org
Arabidopsis lyrata *only for gene family analysis	Hu and colleagues [110]	https://phytozome-next.jgi.doe.gov/info/Alyrata_v2_1
*Azolla filiculoides* Galgenwaard genome	Li and colleagues [111]	https://www.fernbase.org
*Bambusina borreri* CCAC 0045 1KP Code QWFV	Carpenter and colleagues [112]	https://db.cngb.org/onekp/
*Bathycoccus prasinos*	Moreau and colleagues [113]	https://bioinformatics.psb.ugent.be/orcae/overview/Bathy
*Brachypodium distachyon* Bd21	The International *Brachypodium* Initiative [114]	https://phytozome-next.jgi.doe.gov/info/Bdistachyon_v3_1
Brassica oleracea *only for gene family analysis	Liu and colleagues [115]	https://phytozome-next.jgi.doe.gov/info/Boleraceacapitata_v1_0
Brassica rapa *only for gene family analysis	Goodstein and colleagues [116]	https://phytozome-next.jgi.doe.gov/info/BrapaFPsc_v1_3
Capsella grandiflora *only for gene family analysis	Slotte and colleagues [117]	https://phytozome-next.jgi.doe.gov/info/Cgrandiflora_v1_1
Carica papaya *only for gene family analysis	Ming and colleagues [118]	https://phytozome-next.jgi.doe.gov/info/Cpapaya_ASGPBv0_4
Ceratopteris richardii *only for gene family analysis	Marchant and colleagues [119]	https://www.ncbi.nlm.nih.gov/bioproject/?term=PRJNA729743
*Chaetosphaeridium globosum* SAG 26.98	Cooper and Delwiche [120]	https://www.ncbi.nlm.nih.gov/sra/SRR16849197
*Chara braunii* S276	Nishiyama and colleagues [41]	https://bioinformatics.psb.ugent.be/orcae/overview/Chbra
*Chlamydomonas reinhardtii* CC-503	Merchant and colleagues [121] Blaby and colleagues [122]	https://phytozome.jgi.doe.gov/pz/portal.html#!info?alias=Org_Creinhardtii
*Chlorella vulgaris* CCAP 211/11P	Cecchin and colleagues [123]	https://www.ncbi.nlm.nih.gov/Traces/wgs/wgsviewer.cgi?val=SIDB00000000
*Chlorokybus cerfﬁi* SAG 34.98	de Vries and colleagues [44] Irisarri and colleagues [124]	https://datadryad.org/stash/dataset/doi:10.5061/dryad.0gb5mkm25
*Chlorokybus melkonianii* CCAC 0220 *only for gene family analysis	Wang and colleagues [125]	https://db.cngb.org/search/assembly/CNA0002352/
*Closterium lunula* M2156 1KP Code DRFX	Carpenter and colleagues [112]	https://db.cngb.org/onekp/
*Closterium peracerosum-strigosum-littorale* NIES-64 (mt-)	Kawaguchi YQ and colleagues [126]	https://www.ncbi.nlm.nih.gov/sra/?term=ERR10800206 - ERR10800208
*Closterium peracerosum-strigosum-littorale* NIES-65 (mt+)	Kawaguchi YQ and colleagues [126]	https://www.ncbi.nlm.nih.gov/sra/?term=ERR10800221 - ERR10800223
*Closterium peracerosum–strigosum–littorale* NIES-67 *only for gene family analysis	Sekimoto and colleagues [13]	https://www.ncbi.nlm.nih.gov/datasets/genome/GCA_027943415.1/
*Closterium peracerosum–strigosum–littorale* NIES-68 *only for gene family analysis	Sekimoto and colleagues [13]	https://www.ncbi.nlm.nih.gov/datasets/genome/GCA_027943385.1/
*Coccomyxa subellipsoidea* NIES-2166	Blanc and colleagues [127]	https://www.ncbi.nlm.nih.gov/Traces/wgs/wgsviewer.cgi?val=AGSI00000000.1
*Coleochaete scutata* UTEX 2567 (mislabeled as *Coleochaete orbicularis* UTEX 2651) [21]	Cooper and Delwiche [120]	https://figshare.com/articles/dataset/Green_algal_transcriptomes_for_phylogenetics_and_comparative_genomics/1604778
*Coleochaete scutata* SAG 50.90 transcriptome	de Vries and colleagues [44]	https://www.ncbi.nlm.nih.gov/Traces/wgs/wgsviewer.cgi?val=GFXZ00000000
*Cosmarium broomei* CCAC 0143 1KP Code HIDG	Carpenter and colleagues [112]	https://db.cngb.org/onekp/
*Cosmarium crenatum* MZCH-561	Mundt and colleagues [128]	https://www.ncbi.nlm.nih.gov/sra/?term=ERR3005824 and ERR3005825
*Cosmarium granatum* CCAC 0137 1KP Code MNNM	Carpenter and colleagues [112]	https://db.cngb.org/onekp/
*Cosmarium ochthodes* CCAC 1205 1KP Code HJVM	Carpenter and colleagues [112]	https://db.cngb.org/onekp/
*Cosmarium ochthodes* M1384 1KP Code STKJ	Carpenter and colleagues [112]	https://db.cngb.org/onekp/
*Cosmarium subtumidum* CCAC 3067 1KP Code WDGV	Carpenter and colleagues [112]	https://db.cngb.org/onekp/
*Cosmarium tinctum* CCAC 0218 1KP BHBK	Carpenter and colleagues [112]	https://db.cngb.org/onekp/
*Cosmarium turpinii* UTEX 733	Jackrel and colleagues [129]	https://www.ncbi.nlm.nih.gov/sra/?term=SRR3478572, SRR3478573, SRR3478589
*Cosmocladium cf. constrictum* CCAC 0217 1KP WDGV	Carpenter and colleagues [112]	https://db.cngb.org/onekp/
*Cylindrocystis brebissonii* CCAC 0211 1KP YOXI	Carpenter and colleagues [112]	https://db.cngb.org/onekp/
*Cylindrocystis brebissonii* CCAC 2853 1KP YLBK	Carpenter and colleagues [112]	https://db.cngb.org/onekp/
*Cylindrocystis brebissonii* SAG 615-1	This Study	
*Cylindrocystis cushleckae* CCAC 0213 1KP JOJQ	Carpenter and colleagues [112]	https://db.cngb.org/onekp/
*Cylindrocystis sp.* CCAC 0212 1KP code VAZE	Carpenter and colleagues [112]	https://db.cngb.org/onekp/
*Desmidium aptogonum* CCAC 2329 1KP DFDS	Carpenter and colleagues [112]	https://db.cngb.org/onekp/
*Euastrum humerosum var. affine* CCAC 1175 1KP GYRP	Carpenter and colleagues [112]	https://db.cngb.org/onekp/
Gnetum montanum *only for gene family analysis	Wan and colleagues [130]	https://www.ncbi.nlm.nih.gov/bioproject/PRJNA339497/
*Gonatozygon brebissonii* GB138	Su and colleagues [131]	SRR12975768
*Gonatozygon kinahani* CCAC 0216	Carpenter and colleagues [112]	ERR2041121
*Gossypium hirsutum* v2.1 *only for gene family analysis	Li and colleagues [132] Chen and colleagues [133]	https://phytozome-next.jgi.doe.gov/info/Ghirsutum_v2_1
*Isoetes taiwanensis* Kuo4500	Wickell and colleagues [134]	https://genomevolution.org/coge/GenomeInfo.pl?gid=61511 and https://www.ncbi.nlm.nih.gov/bioproject/735564
*Klebsormidium nitens* NIES-2285	Hori and colleagues [135]	https://phycocosm.jgi.doe.gov/Klenit1_1/Klenit1_1.home.html
*Marchantia polymorpha* genome v3.1	Bowman and colleagues [136]	https://phytozome.jgi.doe.gov/pz/portal.html#!info?alias=Org_Mpolymorpha
Maximum likelihood phylogeny (Fig 1)	This Study	https://zenodo.org/records/18392035
*Mesostigma viride* CCAC 1140 and NIES-296	Wang and colleagues [125]	https://db.cngb.org/search/assembly/CNA0002352/
*Mesotaenium caldariorum* SAG 150.80	This Study	
*Mesotaenium caldariorum* SAG 230-1	This Study	
*Mesotaenium caldariorum* SAG 648-1 1KP Code HKZW	Carpenter and colleagues [112]	https://db.cngb.org/onekp/
*Mesotaenium endlicherianum* SAG 12.97 Gene models V2	Cheng and colleagues [10] Dadras and colleagues [90] Rieseberg and colleagues [137]	https://www.ncbi.nlm.nih.gov/bioproject/PRJNA541331/ https://www.ncbi.nlm.nih.gov/sra/?term=SRS17311714, SRS17311718, SRS17311720-SRS17311725
*Mesotaenium kramstae* UTEX 1025 1KP Code NBYP	Carpenter and colleagues [112]	https://db.cngb.org/onekp/
*Mesotaenium pyrenoidosum* SAG 1.88	This Study	
*Micrasterias fimbriata* CCAC 1188 1KP Code MCHJ	Carpenter and colleagues [112]	https://db.cngb.org/onekp/
Micromonas pusilla *only for gene family analysis	Worden and colleagues [138]	https://phycocosm.jgi.doe.gov/MicpuC3v2/MicpuC3v2.home.html
*Micromonas* sp. *only for gene family analysis	Worden and colleagues [138]	https://phycocosm.jgi.doe.gov/MicpuN3v2/MicpuN3v2.home.html
*Mougeotia scalaris* SAG 164.80	Cooper and Delwiche [120]	https://figshare.com/articles/dataset/Green_algal_transcriptomes_for_phylogenetics_and_comparative_genomics/1604778
*Mougeotia scalaris* SAG 164.80 (Labeled wrongly as SAG 164.90)	Cooper and Delwiche [120]	https://www.ncbi.nlm.nih.gov/sra/?term=SRR16849218
*Mougeotia sp.* CCAC 0197	Carpenter and colleagues [112]	https://db.cngb.org/onekp/
*Mougeotia sp.* MZCH 240/ SVCK-240	de Vries and colleagues [89] Fürst-Jansen and colleagues [139]	https://www.ncbi.nlm.nih.gov/bioproject/PRJNA543475/
*Mougeotiopsis calospora* MZCH-580	Hess and colleagues [16]	https://www.ncbi.nlm.nih.gov/sra/SRR19751296
*Netrium digitus* CCAC 0148 1KP Code FFGR	Carpenter and colleagues [112]	https://db.cngb.org/onekp/
*Nicotiana tabacum* TN90 *only for gene family analysis	Sierro and colleagues [140]	https://www.ncbi.nlm.nih.gov/bioproject/PRJNA208209
*Nitella mirabilis* S040	Ju and colleagues [141]	https://www.ncbi.nlm.nih.gov/Traces/wgs/wgsviewer.cgi?val=GBST01&search=GBST01000000&display=scaffolds
*Nucleotaenium eifelense* CCAC 3006 1KP Code KMNX	Carpenter and colleagues [112]	https://db.cngb.org/onekp/
*Onychonema laeve* CCAC 0151 1KP Code GGWH	Carpenter and colleagues [112]	https://db.cngb.org/onekp/
Oryza sativa *only for gene family analysis	Ouyang and colleagues [142]	https://phytozome-next.jgi.doe.gov/info/Osativa_v7_0
Ostreococcus lucimarinus *only for gene family analysis	Palenik and colleagues [143]	https://phycocosm.jgi.doe.gov/Ost9901_3/Ost9901_3.home.html
*Penium exiguum* CCAC 0142 1KP Code YSQT	Carpenter and colleagues [112]	https://db.cngb.org/onekp/
*Penium margaritaceum* SAG 22.82	Cooper and Delwiche [120]	https://www.ncbi.nlm.nih.gov/sra/?term=SRR16849209
*Penium margaritaceum* Skd-8 = SAG 2640	Jiao and colleagues [11]	http://bioinfo.bti.cornell.edu/cgi-bin/Penium/download.cgi
*Phymatodocis nordstedtiana* SAG 47.89 1KP Code RPQV	Carpenter and colleagues [112]	https://db.cngb.org/onekp/
*Physcomitrium patens* Gransden	Lang and colleagues [144] Bi and colleagues [145]	https://phytozome.jgi.doe.gov/pz/portal.html#!info?alias=Org_Ppatens
Picea abies *only for gene family analysis	Nystedt and colleagues [146]	https://www.treegenesdb.org/org/Picea-abies
*Planotaenium ohtanii* CCAC 0202 1KP Code SNOX	Carpenter and colleagues [112]	https://db.cngb.org/onekp/
*Pleurotaenium trabecula* CCAC 0163 1KP Code MOYY	Carpenter and colleagues [112]	https://db.cngb.org/onekp/
Salvinia cucullata *only for gene family analysis	Li and colleagues [111]	https://fernbase.org/ftp/Salvinia_cucullata/
Selaginella moellendorffii *only for gene family analysis	Banks and colleagues [147]	https://phytozome-next.jgi.doe.gov/info/Smoellendorffii_v1_0
*Serritaenia sp.* CCAC 0155 1KP Code WSJO	Carpenter and colleagues [112]	https://db.cngb.org/onekp/
*Serritaenia sp.* SAG 2655	This Study	
*Sirogonium sticticum* UTEX 1985	This Study	
*Solanum lycopersicum* ITAG4.0 *only for gene family analysis	The Tomato Genome Consortium [148]	https://phytozome-next.jgi.doe.gov/info/Slycopersicum_ITAG4_0
Sphagnum fallax *only for gene family analysis	Healey and colleagues [149]	https://phytozome-next.jgi.doe.gov/info/Sfallax_v0_5
*Spirogloea muscicola* CCAC 0214	Cheng and colleagues [10]	https://www.ncbi.nlm.nih.gov/bioproject/PRJNA541068/
*Spirogloea muscicola* CCAC 0214 (OneKP) 1KP Code TPHT	Carpenter and colleagues [112]	https://db.cngb.org/onekp/
*Spirogyra chenii* NIES-4312	This Study	
*Spirogyra chungkingensis* NIES-4302	This Study	
*Spirogyra corrugata* NIES-4303	This Study	
*Spirogyra dentireticulata* NIES-4304	This Study	
*Spirogyra fennica* ACOI-1668	This Study	
*Spirogyra hopeiensis* NIES-4305	This Study	
*Spirogyra longata* NIES-4306	This Study	
*Spirogyra longata* NIES-4307	This Study	
*Spirogyra majuscula* NIES-4308	This Study	
*Spirogyra majuscula* NIES-4309	This Study	
*Spirogyra minuticrassoidea* NIES-4310	This Study	
Spirogyra mirabilis NIES-4311	This Study	
*Spirogyra pratensis* MZCH-10213	de Vries and colleagues [89] Goldbecker and colleagues [15]	https://www.ncbi.nlm.nih.gov/bioproject/PRJNA543475/
*Spirogyra pratensis* UTEX 921	Cooper and Delwiche [120]	https://figshare.com/articles/dataset/Green_algal_transcriptomes_for_phylogenetics_and_comparative_genomics/1604778
*Spirogyra pratensis* UTEX 928	Ju and colleagues [141]	https://www.ncbi.nlm.nih.gov/Traces/wgs/wgsviewer.cgi?val=GBSM01000000
*Spirogyra pseudomaxima* NIES-4313	This Study	
*Spirogyra punctata* NIES-4314	This Study	
*Spirogyra semiornata* NIES-4315	This Study	
*Spirogyra* sp. EDC SP-AU1	Cooper and Delwiche [120]	https://www.ncbi.nlm.nih.gov/sra/?term=SRR16849204
*Spirogyra* sp. CCAC 0205 1KP Code TPHT	Carpenter and colleagues [112]	https://db.cngb.org/onekp/
*Spirogyra* sp. SAG 169.80	This Study	
*Spirogyra* sp. SAG 170.80	This Study	
*Spirogyra tenuissima* ACOI-1925	This Study	
*Spirogyra varians* NIES-4316	This Study	
*Spirotaenia minuta* CCAC 2661 1KP Code NNHQ	Carpenter and colleagues [112]	https://db.cngb.org/onekp/
*Staurastrum punctulatum* SAG 679-1	Jackrel and colleagues [129]	https://www.ncbi.nlm.nih.gov/sra/?term=SRR3478579 -SRR3478581
*Staurastrum sebaldii* CCAC 1129 1KP Code ISHC	Carpenter and colleagues [112]	https://db.cngb.org/onekp/
*Staurastrum tetracerum* SAG 7.94	Paul and colleagues [150]	https://www.ncbi.nlm.nih.gov/sra/?term=SRR1174746
*Staurodesmus convergens* CCAC 2558 1KP Code WCQU	Carpenter and colleagues [112]	https://db.cngb.org/onekp/
*Staurodesmus omearae* CCAC 0751	Carpenter and colleagues [112]	https://db.cngb.org/onekp/
Theobroma cacao *only for gene family analysis	Argout and colleagues [151] Motamayor and colleagues [152]	https://phytozome-next.jgi.doe.gov/info/Tcacao_v1_1
Triticum aestivum *only for gene family analysis	The International Wheat Genome Sequencing Consortium [153]	https://plants.ensembl.org/Triticum_aestivum/Info/Index
*Ulva mutabilis* Føyn	De Clerck and colleagues [154]	https://bioinformatics.psb.ugent.be/orcae/overview/Ulvmu
*Volvox carteri*	Prochnik and colleagues [155]	https://phycocosm.jgi.doe.gov/phycocosm/home/releases?flt=volvox+carteri
*Xanthidium antilopaeum* CCAC 1229 1KP Code GBGT	Carpenter and colleagues.	https://db.cngb.org/onekp/
*Zygnema cylindricum* SAG 698-1a_XF *only for gene family analysis	Feng and colleagues [12]	https://phycocosm.jgi.doe.gov/Zygcyl6981a_1/Zygcyl6981a_1.home.html
*Zygnema circumcarinatum* SAG 698-1b	Feng and colleagues [12] Rieseberg and colleagues [137]	https://phycocosm.jgi.doe.gov/Zygcir6981b_2/Zygcir6981b_2.home.html https://www.ncbi.nlm.nih.gov/sra/?term=SRR24166998 - SRR24167004, SRR24167011, SRR24167014-SRR24167023
*Zygnema circumcarinatum* UTEX 1559 *only for gene family analysis	Feng and colleagues [12]	https://phycocosm.jgi.doe.gov/Zygcir1559_1/Zygcir1559_1.home.html
*Zygnema circumcarinatum* UTEX 1560 *only for gene family analysis	Feng and colleagues [12]	https://phycocosm.jgi.doe.gov/Zygcir1560_1/Zygcir1560_1.home.html
*Zygnema collinsianum* ACOI-1666	This Study	
*Zygnema collinsianum* ACOI-2544	This Study	
*Zygnema cylindricum* SAG 698-2	This Study	
*Zygnema extenue* UTEX 1740	This Study	
*Zygnema frigidum* ACOI-2542	This Study	
*Zygnema irregulare* SAG 2674	This Study	
*Zygnema irregulare* SAG 2675	This Study	
*Zygnema peliosporum* SAG 698-3a	This Study	
*Zygnema peliosporum* SAG 698-3b	This Study	
*Zygnema pseudogedeanum* CCAC 0199	Hess and colleagues [156]	https://www.ncbi.nlm.nih.gov/sra/?term=SRR8869038
*Zygnema pseudogedeanum* SAG 38.96	This Study	
*Zygnema* sp. Alga2O-2499	This Study	
*Zygnema* sp. SAG 2418	This Study	
*Zygnema* sp. SAG 2419	This Study	
*Zygnema* sp. SAG 2642	This Study	
*Zygnema* sp. SAG 698-4	This Study	
*Zygnema* sp. ZygnemaMat	Rippin and colleagues [157]	https://www.ncbi.nlm.nih.gov/sra?linkname=bioproject_sra_all&from_uid=498913
*Zygnema tunetanum* CPCC-136	This Study	
*Zygnemopsis minutum* ACOI-60	This Study	
*Zygnemopsis* sp. CCAP 699/1 1KP Code MFZO	Carpenter and colleagues [112]	https://db.cngb.org/onekp/
Experimental Models: Organisms/Strains		
*Coleochaete scutata* UTEX 610	Bierenbroodspot and colleagues [20,21]	
Software and Algorithms		
ApplyPPPFormat (apply Phylopypruner format)	Bierenbroodspot and colleagues [20,21]	https://github.com/mjbieren/ApplyPPPFormat
BUSCO v.5.0.0	Seppey and colleagues [158]	https://busco.ezlab.org
ClipKIT	Steenwyk and colleagues [159]	https://github.com/JLSteenwyk/ClipKIT
FASTQC	Babraham Institute	www.bioinformatics.babraham.ac.uk/projects/fastqc
FilterPPPResult	Bierenbroodspot and colleagues [20,21]	https://github.com/mjbieren/FilterPPPResult
GPDS (Get Positive DataSet)	Bierenbroodspot and colleagues [20,21]	https://github.com/mjbieren/GPDS
Interproscan v5.77	Jones and colleagues [160]	https://interproscan-docs.readthedocs.io/en/v5/index.html
IQ-Tree v1.5.5 (for gene family analysis)	Nguyen and colleagues [161]	http://www.iqtree.org
IQ-Tree2 v2.2.2.7 (pre-analysis)	Minh and colleagues [162]	
IQ-Tree3 V3.0.1 (for final tree interference)	Wong and colleagues [163]	
MAFFT v7.490	Katoh and Standley [164]	https://mafft.cbrc.jp/alignment/software/
ModelFinder	Kalyaanamoorthy and colleagues [22]	http://www.iqtree.org/ModelFinder/
OrthoFinder v3.1.1	Emms and Kelly [165,166]	https://github.com/OrthoFinder/OrthoFinder
OSG (Orthogroup Sequence Grabber)	Bierenbroodspot and colleagues [20,21]	https://github.com/mjbieren/OrthoGroup_Sequence_Grabber
PAML 4	Yang [167]	http://abacus.gene.ucl.ac.uk/software/#phylogenetic-analysis-by-maximum-likelihood-paml
Phytools	Revell [168]	https://cran.r-project.org/web/packages/phytools/index.html
Posterior Mean Site Frequency Profiles	Wang and colleagues [169]	Implemented in IQ-Tree http://www.iqtree.org
PREQUAL	Whelan and colleagues [170]	https://github.com/simonwhelan/prequal
Tracer v1.7.1	Rambaut and colleagues [171]	https://github.com/beast-dev/tracer/releases/tag/v1.7.1
Transcdecoder v.5.5.0	Brian J. Haas	https://github.com/TransDecoder/TransDecoder/releases
Trimal v1.4.rev15	Capella-Gutierrez and colleagues [172]	http://trimal.cgenomics.org
Trimmomatic v0.36	Bolger and colleagues [173]	http://www.usadellab.org/cms/?page=trimmomatic

### Resource availability

#### Lead contact.

Further information and requests for resources and reagents should be directed to and will be fulfilled by the lead contact, Jan de Vries (devries.jan@uni-goettingen.de).

#### Materials availability.

This study did not generate new unique reagents.

### Experimental model and subject details

#### Algal strains.

Strains were obtained from the Culture Collection of Algae at Göttingen University (SAG) [174], the microbial culture collection of the National Institute for Environmental Studies in Japan (NIES), the Coimbra Collection of Algae (ACOI), the Microalgae and Zygnematophyceae Collection Hamburg (MZCH), and the culture collection of algae at the University of Texas at Austin (UTEX).

### Method details

#### Algal culturing.

All cultures were maintained for six weeks in 3NBBM or MiEB12 liquid medium (medium 26a in Schlösser [175], and medium 7 in Schlösser [176]) under standardized laboratory conditions (18 °C; 14:10 h light:dark cycle; 25–35 μmol photons m^−2^ s^−1^) using full-spectrum fluorescent lighting.

#### Light microscopy.

High-resolution images of the studied strains were obtained with an Olympus BX-60 microscope (Olympus, Japan) with DIC equipped with a ProgRes C14plus camera and the ProgRes CapturePro Software (version 2.9.01) (JENOPTIK AG, Jena, Germany). All investigated strains were examined in week six of cultivation.

#### RNA extraction.

For the RNA extraction of the strains, 50 mL of 6-week-old liquid culture were centrifuged for 5 min at 20 °C and 11,000 rpm and the supernatant was removed. Pellets were transferred into 1.5 ml BioMasherII tubes (Nippi, Japan) and each sample was disrupted using PowerMasherII (Nippi, Japan) after freezing in liquid nitrogen. RNA extraction was done using the Spectrum Plant Total RNA Kit (Sigma-Aldrich Chemie GmbH, Germany) according to the manufacturer’s instructions. DNAse I treatment (Thermo Fisher, Waltham, MA, USA) was applied to the RNA samples, and their integrity and concentration were assessed using a 1% agarose gel with a Midora green stain, and Nanodrop (Thermo Fisher), respectively. The RNA samples were shipped on dry ice to Novogene (Munich, Germany).

#### RNA sequencing, transcriptome assembly, and transcriptome data.

At Novogene (Munich, Germany), RNA quality was verified using a Bioanalyzer (Agilent Technologies, USA). Libraries were prepared using poly-A enrichment and directional mRNA library construction protocols. After the libraries were quality-checked, sequencing was conducted on an Illumina NovaSeq 6000 platform using dual-indexed Novogene adapters: 5′- AGATCGGAAGAGCGTCGTGTAGGGAAAGAGTGTAGATCTCGGTGGTCGCCGTATCATT-3′ for read 1 and 5′- GATCGGAAGAGCACACGTCTGAACTCCAGTCACGGATGACTATCTCGTATGCCGTCTTCTGCTTG-3′.

We conducted transcriptome sequencing on 43 isolates belonging to different orders of the Zygnematophyceae. All transcriptomes were assembled de novo with Trinity [177] v2.15.1, following adapter trimming with Trimmomatic [173] (--trimmomatic “ILLUMINACLIP:novogene_adapter_sequences.fa:2:30:10:2:keepBothReads LEADING:3 TRAILING:3 MINLEN:36″) and default settings. Splicing isoforms were collapsed into SuperTranscripts [178] using Trinity’s implementation. Completeness was assessed with BUSCO [158] v5.4.3 using the ‘eukaryota_odb10′ dataset, yielding an average completeness of 94.84%. Protein-coding sequences were predicted with TransDecoder v5.5.0, retaining only the longest open reading frame per transcript (--single_best_only).

#### Phylogenomic analysis: Preamble.

Phylogenomic analyses and ancestral character state reconstruction were conducted following the framework outlined in Bierenbroodspot and colleagues [20], with the following methodological refinements. For orthogroup selection, we utilized only the Phylogenetic Hierarchical Orthogroups at the tree root (N0.tsv file produced by OrthoFinder v3.1.1 [165,166]) in conjunction with Orthogroup Sequence Grabber (OSG) [20,21]. A streamlined version of the COGS software [20,21] was implemented, excluding one redundant MAFFT [164]/IQ-TREE [161–163] alignment step. Similarly, the final ClipKit [159] trimming step was omitted, as it did not enhance alignment quality under the revised workflow.

#### Phylogenomic analysis: Dataset construction for phylotranscriptomics.

To eliminate contamination from de novo transcriptome assemblies, sequence similarity searches were conducted using the MMseqs2 tool [179] against a custom database containing proteins from the axenic strains MZCH-10213, SAG 1.88, SAG 12.97, SAG 615-1, SAG 698-1a, and SAG 698-1b as positive control, and potential contaminants including RefSeq representative genomes of bacteria (43,588), fungi (1,225), invertebrate animals (7,213), virus (13,134), archaea (1,120), protists (893), mitochondrial genes (240,117), plasmids (4,710), and plastids (12,307) (downloaded July 11, 2023). An iterative approach was used for MMseqs2 (--start-sens 1 --sens-steps 3 -s 7 --alignment-mode 3 --max-seqs 10), and decontamination was completed with GPDS [20,21]. Only sequences whose best match was against the combined positive set were retained. Potential contaminant-matching sequences were archived and are available via Zenodo https://doi.org/10.5281/zenodo.18392035

#### Phylogenomic analysis: Phylotranscriptomic analysis.

Orthogroup inference was performed using OrthoFinder [166] v3.1.1 with a species tree following the framework of the One Thousand Plant Transcriptomes Initiative [2], including chlorophytes, *Chlorokybus cerffii* SAG 34.98, *Mesostigma viride* NIES-296, and *Klebsormidium nitens* NIES-2285, alongside multiple Phragmoplastophyta and Coleochaetophyceae (see “09_Orthofinder.zip” on Zenodo). A total of 60,932 orthogroups were identified. Two orthogroup subsets were generated using OSG [20,21]: one enriched for ingroup taxa (13,409 orthogroups, filter: 2 of 4 groups) and another for outgroups (7,253 orthogroups, filter: 10 of 17 groups). All data are available at Zenodo (“10_OSG.zip”).

Multiple sequence alignments were performed with MAFFT [164] v7.304 under default settings. Maximum likelihood trees were constructed using the IQ-TREE 2 software [162] v2.2.2.7 with automated model selection via ModelFinder [22] (selection of best-fit models was based on Bayesian Information Criterion), using SH-like aLRT (-alrt 1000) and ultrafast bootstrap replicates (-bb 1000). Tree files were reformatted for PhyloPyPruner v1.2.4 using ApplyPPPFormat (https://github.com/mjbieren/ApplyPPPFormat). PhyloPyPruner (https://github.com/fethalen/phylopypruner) was used to remove paralogs. For the orthogroup subset biased towards the ingroup, we used the following settings: --mask pdist --prune MI --min-taxa 3 --trim-lb 5 --min-support 0.75 --threads 50 --trim-divergent 1.25 --jackknife, yielding 93,280 ortholog alignments. For the outgroup-centered subset, we used: --min-taxa 10 --min-gene-occupancy 0.1, yielding 5,960ortholog alignments. After taxonomic filtering to ensure a balanced taxonomic representation in alignments (FilterPPPResult, -t 10 (of 110)), we retained 17,588 ingroup-rich and (FilterPPPResult, -t 2) 5,923 outgroup-rich loci. These were merged using COGS [21] and then pruned to 5,225 unique orthogroups. A final PhyloPyPruner run produced 80,973 loci; after filtering (FilterPPPResult, -t 30), 2,243 loci remained. These were masked using PREQUAL [170] v1.02 and re-aligned with MAFFT (G-INS-i, ‘--allowshift --unalignlevel 0.8′). Gappy columns (>75% gaps) were removed using ClipKIT [159] v2.0.1. The final concatenated matrix was created using phyx [180] comprising 126 taxa, 2,243 loci, and 579,537 aligned amino acid sites. Phylogenies were reconstructed with IQ-TREE 3 V3.0.1, using LG+C60+F+G-PMSF, 1000 replicates of each SH-aLRT and UFBoot [181].

#### Ancestral character state reconstruction.

Ancestral character state reconstruction (ACSR) estimates the probability of character states at ancestral nodes from the observed states at the terminal taxa, the phylogenetic tree, including branch lengths, and an explicit model of character evolution. We used an equal-rates model, assigning an identical transition rate of 1 to all possible state transitions, i.e., analyses assumed symmetric transition rates between unordered states. ACSR was conducted using Phytools [168], employing Yang’s re-rooting method [182]. Traits were coded with a 5-state model: (1) unicellular, (2) multicellular, (3) Package-like, (4) *Bona fide* filaments, and (5) Chain-like filaments.

#### Gene family analysis.

For gene family analysis, we worked with a dataset of predicted proteins derived from (i) the transcriptomes generated in this study and two available ones and (ii) published genomes of phylodiverse Chloroplastida (see Resource table). We searched for homologs of the genes of interest using representative sequences from *Arabidopsis* as a query in a BLASTp search against the full protein dataset. An e-value cutoff of 10^-7^ was applied; in case of large gene families, the number of species was reduced. All sequences were aligned using MAFFT [164] v7.490 (L-INS-i) and a maximum likelihood phylogeny was computed using IQ-TREE [161] with 1000 UFBoot [183] pseudoreplicates and best-fit models chosen according to Bayesian Information Criterion by ModelFinder [22]. The AHK gene family tree was annotated using PFAM domains predicted by InterProScan [160] v5.77-108.0. The domains were consolidated per clade. A clade was annotated to contain a domain when it appeared in several proteins within that clade. Outlier domains, e.g., only predicted for 1 protein within the whole clade, were ignored as an observation based on a single protein (*n* = 1) provides very limited support for drawing conclusions about the broader evolutionary pattern within that clade. We therefore adopted a conservative filtering criterion and retained only labels for clades in which the domain was detected in more than one protein, thereby reducing the influence of potentially spurious individual observations on the overall analysis. We summarized PFAM PAS fold and PAS domain annotations (PF00989, PF08447, PF08448, PF13188, PF13426) with one symbol.

#### Overview of the quantifications and statistical analysis.

The final maximum likelihood phylogenomic tree was inferred using the LG + C60 model with 1000 SH-aLRT and 1000 ultrafast bootstrap replicates. Additional analyses incorporating site-heterogeneous PMSF models were conducted using a guide tree inferred with LG + C60 + F + Γ. These consistently recovered the same topology as the original LG + C60-based tree.

ModelFinder [22] identified LG + C60 as the best -fitting model for protein evolution compared to the more standard empirical amino acid substitution model with site-rate heterogeneity LG + I + G4, as indicated by improved likelihood scores and a lower Bayesian Information Criterion (LG + C60: Log(Likelihood) = −15,486,259.27; Bayesian Information Criterion = 30,975,836.031; LG + I + G4: Log(Likelihood) = −15,683,898.390; Bayesian Information Criterion = 31,371,127.541). The maximum likelihood phylogenomic tree was inferred under the LG + C60 model using 1,000 SH-aLRT and 1,000 ultrafast bootstrap replicates. To account for site heterogeneity, an additional analysis was performed using the PMSF approximation with LG + C60 + F + Γ, guided by the previously inferred tree. This analysis consistently recovered the same tree topology, reinforcing the robustness of the phylogenetic inference across model frameworks.

For gene family analyses, 144 protein models were tested using ModelFinder [22] and Bayesian Information Criterion was used to determine the best-fit model. 1000 UFBoot [183] pseudoreplicates were computed per gene tree.

## Supporting information

S1 Fig Fully labeled phylogenetic trees of signature gene families. Related to Fig 3. Fully labeled phylogenies shown in Fig 3. The data and code needed to generate this Figure can be found in https://zenodo.org/records/18392035 as well as https://doi.org/10.5281/zenodo.22172294 on Zenodo.
(PDF)

## Abbreviations

ABA: Abscisic acid
ACSR: ancestral character state reconstruction
AHK: Arabidopsis histidine kinases
CAD: cinnamyl alcohol dehydrogenase
CIDAS: Campus Institute Data Science
LCA: last common ancestor
MZCH: Microalgae and Zygnematophyceae Collection Hamburg
NIES: National Institute for Environmental Studies in Japan
OSG: Orthogroup Sequence Grabber
PMSF: Posterior Mean Site Frequency
PP: posterior probabilities
SADs: sinapyl alcohol dehydrogenases
TAIR: The Arabidopsis Information Resource

## References

[1] Wickett NJ, Mirarab S, Nguyen N, Warnow T, Carpenter E, Matasci N, et al. Phylotranscriptomic analysis of the origin and early diversification of land plants. Proc Natl Acad Sci U S A. 2014;111(45):E4859-68. doi: 10.1073/pnas.1323926111 25355905 PMC4234587

[2] One Thousand Plant Transcriptomes I. One thousand plant transcriptomes and the phylogenomics of green plants. Nature. 2019;574(7780):679–85. Epub 20191023. doi: 10.1038/s41586-019-1693-2 31645766 PMC6872490

[3] Puttick MN, Morris JL, Williams TA, Cox CJ, Edwards D, Kenrick P, et al. The Interrelationships of land plants and the nature of the ancestral embryophyte. Curr Biol. 2018;28(5):733-745.e2. doi: 10.1016/j.cub.2018.01.063 29456145

[4] Donoghue PCJ, Harrison CJ, Paps J, Schneider H. The evolutionary emergence of land plants. Curr Biol. 2021;31(19):R1281–98. doi: 10.1016/j.cub.2021.07.038 34637740

[5] Bowles AMC, Williamson CJ, Williams TA, Lenton TM, Donoghue PCJ. The origin and early evolution of plants. Trends Plant Sci. 2023;28(3):312–29. doi: 10.1016/j.tplants.2022.09.009 36328872

[6] Baştürk MN, Kunz CF, Darienko T, Pröschold T, de Vries J, Chen H. Phylogenomic frameworks from deep archaeplastid evolution to embryophyte diversification. Curr Biol. 2026;36(11):R522–44. doi: 10.1016/j.cub.2026.03.019 42259268

[7] Goldbecker ES, de Vries J. Systems biology of streptophyte cell evolution. Annu Rev Plant Biol. 2025;76(1):493–522. doi: 10.1146/annurev-arplant-083123-060254 39819561

[8] Carrillo-Carrasco VP, Hernandez-Garcia J, Girou C, Grubor I, Keller J, Lim E, et al. A roadmap to developing unified streptophyte algal model systems. Curr Biol. 2025;35(14):R725–38. doi: 10.1016/j.cub.2025.05.023 40695244

[9] Ohtaka K, Sekimoto H. Zygnematophycean algae: Possible models for cellular and evolutionary biology. Semin Cell Dev Biol. 2023;134:59–68. doi: 10.1016/j.semcdb.2022.03.042 35430142

[10] Cheng S, Xian W, Fu Y, Marin B, Keller J, Wu T, et al. Genomes of subaerial Zygnematophyceae provide insights into land plant evolution. Cell. 2019;179(5):1057-1067.e14. doi: 10.1016/j.cell.2019.10.019 31730849

[11] Jiao C, Sørensen I, Sun X, Sun H, Behar H, Alseekh S, et al. The *Penium margaritaceum* genome: Hallmarks of the origins of land plants. Cell. 2020;181(5):1097-1111.e12. doi: 10.1016/j.cell.2020.04.019 32442406

[12] Feng X, Zheng J, Irisarri I, Yu H, Zheng B, Ali Z, et al. Genomes of multicellular algal sisters to land plants illuminate signaling network evolution. Nat Genet. 2024;56(5):1018–31. doi: 10.1038/s41588-024-01737-3 38693345 PMC11096116

[13] Sekimoto H, Komiya A, Tsuyuki N, Kawai J, Kanda N, Ootsuki R, et al. A divergent RWP-RK transcription factor determines mating type in heterothallic Closterium. New Phytol. 2023;237(5):1636–51. doi: 10.1111/nph.18662 36533897

[14] Kunz CF, Goldbecker ES, de Vries J. Functional genomic perspectives on plant terrestrialization. Trends Genet. 2025;41(7):617–29. doi: 10.1016/j.tig.2025.02.006 40155238

[15] Goldbecker ES, Varshney D, Holzhausen A, Darienko T, Zhou H, Dadras A, et al. The *Spirogyra pratensis* genome illuminates the evolution of developmental programs and spiral chloroplast biology. Plant Cell. 2026;38(8):koag224. doi: 10.1093/plcell/koag224 42520407 PMC13519894

[16] Hess S, Williams SK, Busch A, Irisarri I, Delwiche CF, de Vries S, et al. A phylogenomically informed five-order system for the closest relatives of land plants. Curr Biol. 2022;32(20):4473-4482.e7. doi: 10.1016/j.cub.2022.08.022 36055238 PMC9632326

[17] Round FE. The taxonomy of the Chlorophyta. II. Br Phycol J. 1971;6(2):235–64. doi: 10.1080/00071617100650261

[18] Guiry MD. Taxonomy and nomenclature of the Conjugatophyceae (= Zygnematophyceae). ALGAE. 2013;28(1):1–29. doi: 10.4490/algae.2013.28.1.001

[19] van den Hoek C, Mann DG, Jahns HM. Algae: An introduction to phycology; 1995.

[20] Bierenbroodspot MJ, Darienko T, de Vries S, Fürst-Jansen JMR, Buschmann H, Pröschold T, et al. Phylogenomic insights into the first multicellular streptophyte. Curr Biol. 2024;34(3):670-681.e7. doi: 10.1016/j.cub.2023.12.070 38244543 PMC10849092

[21] Bierenbroodspot MJ, Darienko T, de Vries S, Delwiche CF, Lorenz M, Ali Z, et al. Phylogenomics unveil a recent origin of morphological complexity in Coleochaetophyceae. Curr Biol. 2025;35(20):5071-5080.e7. doi: 10.1016/j.cub.2025.08.046 40967205

[22] Kalyaanamoorthy S, Minh BQ, Wong TKF, von Haeseler A, Jermiin LS. ModelFinder: Fast model selection for accurate phylogenetic estimates. Nat Methods. 2017;14(6):587–9. doi: 10.1038/nmeth.4285 28481363 PMC5453245

[23] Wang H-C, Minh BQ, Susko E, Roger AJ. Modeling site heterogeneity with posterior mean site frequency profiles accelerates accurate phylogenomic estimation. Syst Biol. 2018;67(2):216–35. doi: 10.1093/sysbio/syx068 28950365

[24] Stancheva R, Lewis LA, Hall J, Šoljaková T, Permann C, Holzinger A. Genetic and morphological variation in the genus *Zygogonium* (Zygnematophyceae, Charophyta) from localities in Europe and North America and description of *Z. angustum*, sp. nov. J Phycol. 2025;61(3):587–606. doi: 10.1111/jpy.70012 40203186 PMC12168101

[25] Busch A, Hess S. A diverse group of underappreciated zygnematophytes deserves in-depth exploration. Appl Phycol. 2022;3(1):306–23. doi: 10.1080/26388081.2022.2081819

[26] Bowles AMC, Williams TA, Donoghue PC, Campbell DA, Williamson CJ. Metagenome-assembled genome of the glacier alga *Ancylonema* yields insights into the evolution of streptophyte life on ice and land. New Phytol. 2024. 10.1111/nph.19860 38840553

[27] Kunz CF, Goldbecker ES, Darienko T, de Vries J. Genome evolution: Zygnematophyceae on ice. New Phytol. 2024;244(4):1125–7. doi: 10.1111/nph.19960 39001590

[28] Delaux P-M, Radhakrishnan GV, Jayaraman D, Cheema J, Malbreil M, Volkening JD, et al. Algal ancestor of land plants was preadapted for symbiosis. Proc Natl Acad Sci U S A. 2015;112(43):13390–5. doi: 10.1073/pnas.1515426112 26438870 PMC4629359

[29] Kadlubowska JZ. Conjugatophyceae I. Chlorophyta VIII. Zygnematales. In: Ettl H, Gerloff J, Heynig H, Mollenhauer D, editors. Stuttgart (Germany): Gustav-Fischer-Verlag.;1984.

[30] Takano T, Higuchi S, Ikegaya H, Matsuzaki R, Kawachi M, Takahashi F, et al. Identification of 13 *Spirogyra* species (Zygnemataceae) by traits of sexual reproduction induced under laboratory culture conditions. Sci Rep. 2019;9(1):7458. doi: 10.1038/s41598-019-43454-6 31123266 PMC6533312

[31] Permann C, Herburger K, Felhofer M, Gierlinger N, Lewis LA, Holzinger A. Induction of conjugation and zygospore cell wall characteristics in the alpine *Spirogyra mirabilis* (Zygnematophyceae, Charophyta): Advantage under climate change scenarios?. Plants (Basel). 2021;10(8). doi: 10.3390/plants10081740 34451785 PMC8402014

[32] Chen C, Barfuss MHJ, Pröschold T, Schagerl M. Hidden genetic diversity in the green alga *Spirogyra* (Zygnematophyceae, Streptophyta). BMC Evol Biol. 2012;12:77. doi: 10.1186/1471-2148-12-77 22655677 PMC3527229

[33] Permann C, Holzinger A. Zygospore formation in Zygnematophyceae predates several land plant traits. Philos Trans R Soc Lond B Biol Sci. 2024;379(1914):20230356. doi: 10.1098/rstb.2023.0356 39343014 PMC11449217

[34] Pfeifer L, Utermöhlen J, Happ K, Permann C, Holzinger A, von Schwartzenberg K, et al. Search for evolutionary roots of land plant arabinogalactan-proteins in charophytes: Presence of a rhamnogalactan-protein in *Spirogyra pratensis* (Zygnematophyceae). Plant J. 2022;109(3):568–84. doi: 10.1111/tpj.15577 34767672 PMC7612518

[35] Stancheva R, Sheath RG, Hall JD. Systematics of the genus *Zygnema* (Zygnematophyceae, Charophyta) from Californian watersheds(1). J Phycol. 2012;48(2):409–22. Epub 20120319. doi: 10.1111/j.1529-8817.2012.01127.x 27009731

[36] Herburger K, Lewis LA, Holzinger A. Photosynthetic efficiency, desiccation tolerance and ultrastructure in two phylogenetically distinct strains of alpine *Zygnema* sp. (Zygnematophyceae, Streptophyta): Role of pre-akinete formation. Protoplasma. 2015;252(2):571–89. doi: 10.1007/s00709-014-0703-3 25269628 PMC4335129

[37] Permann C, Pichrtová M, Šoljaková T, Herburger K, Jouneau P-H, Uwizeye C, et al. 3D-reconstructions of zygospores in *Zygnema vaginatum* (Charophyta) reveal details of cell wall formation, suggesting adaptations to extreme habitats. Physiol Plant. 2023;175(4):e13988. doi: 10.1111/ppl.13988 37616005 PMC10953328

[38] Pichrtová M, Holzinger A, Kulichová J, Ryšánek D, Šoljaková T, Trumhová K, et al. Molecular and morphological diversity of *Zygnema* and *Zygnemopsis* (Zygnematophyceae, Streptophyta) from Svalbard (High Arctic). Eur J Phycol. 2018;53(4):492–508. doi: 10.1080/09670262.2018.1476920 30487730 PMC6235541

[39] Garduño-Solórzano G, Martínez-García M, Scotta Hentschke G, Lopes G, Castelo Branco R, Vasconcelos VMO, et al. The phylogenetic placement of *Temnogametum* (Zygnemataceae) and description of *Temnogametum iztacalense* sp. nov., from a tropical high mountain lake in Mexico. Eur J Phycol. 2021;56(2):159–73. doi: 10.1080/09670262.2020.1789226

[40] Feng X, Holzinger A, Permann C, Anderson D, Yin Y. Characterization of two *Zygnema* strains (*Zygnema circumcarinatum* SAG 698-1a and SAG 698-1b) and a rapid method to estimate nuclear genome size of zygnematophycean green algae. Front Plant Sci. 2021;12:610381. doi: 10.3389/fpls.2021.610381 33643345 PMC7902510

[41] Nishiyama T, Sakayama H, de Vries J, Buschmann H, Saint-Marcoux D, Ullrich KK, et al. The Chara genome: Secondary complexity and implications for plant terrestrialization. Cell. 2018;174(2):448-464.e24. doi: 10.1016/j.cell.2018.06.033 30007417

[42] Darienko T, Kunz CF, Goldbecker ES, Endriulaitytė E, Bierenbroodspot MJ, Lorenz M, et al. The evolutionary origins of streptophyte multicellularity. New Phytol. 2026;251(5):2365–83. doi: 10.1111/nph.71415 42426897 PMC13443282

[43] Wodniok S, Brinkmann H, Glöckner G, Heidel AJ, Philippe H, Melkonian M, et al. Origin of land plants: Do conjugating green algae hold the key?. BMC Evol Biol. 2011;11:104. doi: 10.1186/1471-2148-11-104 21501468 PMC3088898

[44] de Vries J, Curtis BA, Gould SB, Archibald JM. Embryophyte stress signaling evolved in the algal progenitors of land plants. Proc Natl Acad Sci U S A. 2018;115(15):E3471–80. doi: 10.1073/pnas.1719230115 29581286 PMC5899452

[45] Chen K, Li G-J, Bressan RA, Song C-P, Zhu J-K, Zhao Y. Abscisic acid dynamics, signaling, and functions in plants. J Integr Plant Biol. 2020;62(1):25–54. doi: 10.1111/jipb.12899 31850654

[46] Cutler SR, Rodriguez PL, Finkelstein RR, Abrams SR. Abscisic acid: Emergence of a core signaling network. Annu Rev Plant Biol. 2010;61:651–79. doi: 10.1146/annurev-arplant-042809-112122 20192755

[47] Nishimura N, Sarkeshik A, Nito K, Park S-Y, Wang A, Carvalho PC, et al. PYR/PYL/RCAR family members are major in-vivo ABI1 protein phosphatase 2C-interacting proteins in *Arabidopsis*. Plant J. 2010;61(2):290–9. doi: 10.1111/j.1365-313X.2009.04054.x 19874541 PMC2807913

[48] Melcher K, Ng L-M, Zhou XE, Soon F-F, Xu Y, Suino-Powell KM, et al. A gate-latch-lock mechanism for hormone signalling by abscisic acid receptors. Nature. 2009;462(7273):602–8. doi: 10.1038/nature08613 19898420 PMC2810868

[49] Miyazono K-I, Miyakawa T, Sawano Y, Kubota K, Kang H-J, Asano A, et al. Structural basis of abscisic acid signalling. Nature. 2009;462(7273):609–14. doi: 10.1038/nature08583 19855379

[50] Park S-Y, Fung P, Nishimura N, Jensen DR, Fujii H, Zhao Y, et al. Abscisic acid inhibits type 2C protein phosphatases via the PYR/PYL family of START proteins. Science. 2009;324(5930):1068–71. doi: 10.1126/science.1173041 19407142 PMC2827199

[51] Santiago J, Dupeux F, Round A, Antoni R, Park S-Y, Jamin M, et al. The abscisic acid receptor PYR1 in complex with abscisic acid. Nature. 2009;462(7273):665–8. doi: 10.1038/nature08591 19898494

[52] Yin P, Fan H, Hao Q, Yuan X, Wu D, Pang Y, et al. Structural insights into the mechanism of abscisic acid signaling by PYL proteins. Nat Struct Mol Biol. 2009;16(12):1230–6. doi: 10.1038/nsmb.1730 19893533

[53] Sun Y, Harpazi B, Wijerathna-Yapa A, Merilo E, de Vries J, Michaeli D, et al. A ligand-independent origin of abscisic acid perception. Proc Natl Acad Sci U S A. 2019;116(49):24892–9. doi: 10.1073/pnas.1914480116 31744875 PMC6900503

[54] Zimran G, Shpilman M, Hobson E, Kamisugi Y, Baichman-Kass A, Zhang H, et al. Abscisic acid receptors functionally converge across 500 million years of land plant evolution. Curr Biol. 2025;35(4):818-830.e4. doi: 10.1016/j.cub.2024.12.043 39892384

[55] Zegers JMS, Irisarri I, de Vries S, de Vries J. Evolving circuitries in plant signaling cascades. J Cell Sci. 2024;137(17):jcs261712. doi: 10.1242/jcs.261712 39239891

[56] Zegers JMS, Garcia Ramirez GX, Harzen A, Stolze SC, Salem M, Ziplys A, et al. Homologous ABA-independent kinase tracks coalesced into osmotic stress circuits during plant terrestrialization. bioRxiv. 2026. doi: 10.64898/2026.05.21.726866

[57] Carrillo-Carrasco VP, Hernandez-Garcia J, Mutte SK, Weijers D. The birth of a giant: Evolutionary insights into the origin of auxin responses in plants. EMBO J. 2023;42(6):e113018. doi: 10.15252/embj.2022113018 36786017 PMC10015382

[58] Kuhn A, Roosjen M, Mutte S, Dubey SM, Carrillo Carrasco VP, Boeren S, et al. RAF-like protein kinases mediate a deeply conserved, rapid auxin response. Cell. 2024;187(1):130-148.e17. doi: 10.1016/j.cell.2023.11.021 38128538 PMC10783624

[59] Hernández-García J, Carrillo-Carrasco VP, Rienstra J, Tanaka K, de Roij M, Dipp-Álvarez M, et al. Evolutionary origins and functional diversification of Auxin Response Factors. Nat Commun. 2024;15(1):10909. doi: 10.1038/s41467-024-55278-8 39738167 PMC11685440

[60] Carrillo-Carrasco VP, van Galen M, Bronkhorst J, Mutte S, Kohlen W, Sprakel J, et al. Auxin and tryptophan trigger common responses in the streptophyte alga *Penium margaritaceum*. Curr Biol. 2025;35(9):2078-2087.e4. doi: 10.1016/j.cub.2025.03.037 40209711 PMC12061043

[61] de Roij M, Hernández García J, Das S, Borst JW, Weijers D. ARF degradation defines a deeply conserved step in auxin response. Nat Plants. 2025;11(4):717–24. doi: 10.1038/s41477-025-01975-1 40216983 PMC12014493

[62] Rienstra J, Carrillo-Carrasco VP, de Roij M, Hernandez-Garcia J, Weijers D. A conserved ARF-DNA interface underlies auxin-triggered transcriptional response. Proc Natl Acad Sci U S A. 2025;122(14):e2501915122. doi: 10.1073/pnas.2501915122 40168121 PMC12002309

[63] Endriulaityte E, Kunz CF, Zegers JMS, de Vries J. Streptophyte evolution: Auxin response born out of sensing tryptophan. Curr Biol. 2025;35(9):R333–R6. doi: 10.1016/j.cub.2025.03.017 40328218

[64] Dixon RA, Paiva NL. Stress-induced phenylpropanoid metabolism. Plant Cell. 1995;7(7):1085–97. doi: 10.1105/tpc.7.7.1085 12242399 PMC160915

[65] Dixon RA, Achnine L, Kota P, Liu C-J, Reddy MSS, Wang L. The phenylpropanoid pathway and plant defence-a genomics perspective. Mol Plant Pathol. 2002;3(5):371–90. doi: 10.1046/j.1364-3703.2002.00131.x 20569344

[66] Dixon RA, Barros J. Lignin biosynthesis: old roads revisited and new roads explored. Open Biol. 2019;9(12):190215. doi: 10.1098/rsob.190215 31795915 PMC6936255

[67] Vanholme R, De Meester B, Ralph J, Boerjan W. Lignin biosynthesis and its integration into metabolism. Curr Opin Biotechnol. 2019;56:230–9. doi: 10.1016/j.copbio.2019.02.018 30913460

[68] Emiliani G, Fondi M, Fani R, Gribaldo S. A horizontal gene transfer at the origin of phenylpropanoid metabolism: A key adaptation of plants to land. Biol Direct. 2009;4:7. doi: 10.1186/1745-6150-4-7 19220881 PMC2657906

[69] Labeeuw L, Martone PT, Boucher Y, Case RJ. Ancient origin of the biosynthesis of lignin precursors. Biol Direct. 2015;10:23. doi: 10.1186/s13062-015-0052-y 25994183 PMC4455696

[70] de Vries J, de Vries S, Slamovits CH, Rose LE, Archibald JM. How embryophytic is the biosynthesis of phenylpropanoids and their derivatives in streptophyte algae?. Plant Cell Physiol. 2017;58(5):934–45. doi: 10.1093/pcp/pcx037 28340089

[71] Renault H, Alber A, Horst NA, Basilio Lopes A, Fich EA, Kriegshauser L, et al. A phenol-enriched cuticle is ancestral to lignin evolution in land plants. Nat Commun. 2017;8:14713. doi: 10.1038/ncomms14713 28270693 PMC5344971

[72] Renault H, Werck-Reichhart D, Weng J-K. Harnessing lignin evolution for biotechnological applications. Curr Opin Biotechnol. 2019;56:105–11. doi: 10.1016/j.copbio.2018.10.011 30439673

[73] Kunz CF, de Vries S, de Vries J. Plant terrestrialization: an environmental pull on the evolution of multi-sourced streptophyte phenolics. Philos Trans R Soc Lond B Biol Sci. 2024;379(1914):20230358. doi: 10.1098/rstb.2023.0358 39343031 PMC11528360

[74] de Vries S, Fürst-Jansen JMR, Irisarri I, Dhabalia Ashok A, Ischebeck T, Feussner K, et al. The evolution of the phenylpropanoid pathway entailed pronounced radiations and divergences of enzyme families. Plant J. 2021;107(4):975–1002. doi: 10.1111/tpj.15387 34165823

[75] Mansell RL, Gross GG, Stöckigt J, Franke H, Zenk MH. Purification and properties of cinnamyl alcohol dehydrogenase from higher plants involved in lignin biosynthesis. Phytochemistry. 1974;13(11):2427–35. doi: 10.1016/s0031-9422(00)86917-4

[76] Pan H, Zhou R, Louie GV, Mühlemann JK, Bomati EK, Bowman ME, et al. Structural studies of cinnamoyl-CoA reductase and cinnamyl-alcohol dehydrogenase, key enzymes of monolignol biosynthesis. Plant Cell. 2014;26(9):3709–27. doi: 10.1105/tpc.114.127399 25217505 PMC4213152

[77] Guo D-M, Ran J-H, Wang X-Q. Evolution of the Cinnamyl/Sinapyl Alcohol Dehydrogenase (CAD/SAD) gene family: The emergence of real lignin is associated with the origin of Bona Fide CAD. J Mol Evol. 2010;71(3):202–18. doi: 10.1007/s00239-010-9378-3 20721545

[78] Brill EM, Abrahams S, Hayes CM, Jenkins CL, Watson JM. Molecular characterisation and expression of a wound-inducible cDNA encoding a novel cinnamyl-alcohol dehydrogenase enzyme in lucerne (*Medicago sativa* L.). Plant Mol Biol. 1999;41(2):279–91. doi: 10.1023/a:1006381630494 10579494

[79] Deng W-W, Zhang M, Wu J-Q, Jiang Z-Z, Tang L, Li Y-Y, et al. Molecular cloning, functional analysis of three cinnamyl alcohol dehydrogenase (CAD) genes in the leaves of tea plant, *Camellia sinensis*. J Plant Physiol. 2013;170(3):272–82. doi: 10.1016/j.jplph.2012.10.010 23228629

[80] Jiang S, Tian X, Huang X, Xin J, Yan H. Physcomitrium patens CAD1 has distinct roles in growth and resistance to biotic stress. BMC Plant Biol. 2022;22(1):518. doi: 10.1186/s12870-022-03892-3 36344936 PMC9641914

[81] Chao N, Huang S, Kang X, Yidilisi K, Dai M, Liu L. Systematic functional characterization of cinnamyl alcohol dehydrogenase family members revealed their functional divergence in lignin biosynthesis and stress responses in mulberry. Plant Physiol Biochem. 2022;186:145–56. doi: 10.1016/j.plaphy.2022.07.008 35849944

[82] Kim S-J, Kim M-R, Bedgar DL, Moinuddin SGA, Cardenas CL, Davin LB, et al. Functional reclassification of the putative cinnamyl alcohol dehydrogenase multigene family in *Arabidopsis*. Proc Natl Acad Sci U S A. 2004;101(6):1455–60. doi: 10.1073/pnas.0307987100 14745009 PMC341741

[83] Jun S-Y, Walker AM, Kim H, Ralph J, Vermerris W, Sattler SE, et al. The enzyme activity and substrate specificity of two major cinnamyl alcohol dehydrogenases in Sorghum (*Sorghum bicolor*), SbCAD2 and SbCAD4. Plant Physiol. 2017;174(4):2128–45. doi: 10.1104/pp.17.00576 28606901 PMC5543964

[84] Bukh C, Nord-Larsen PH, Rasmussen SK. Phylogeny and structure of the cinnamyl alcohol dehydrogenase gene family in *Brachypodium distachyon*. J Exp Bot. 2012;63(17):6223–36. doi: 10.1093/jxb/ers275 23028019 PMC3481213

[85] Youn B, Camacho R, Moinuddin SGA, Lee C, Davin LB, Lewis NG, et al. Crystal structures and catalytic mechanism of the *Arabidopsis* cinnamyl alcohol dehydrogenases AtCAD5 and AtCAD4. Org Biomol Chem. 2006;4(9):1687–97. doi: 10.1039/b601672c 16633561

[86] Park HL, Kim TL, Bhoo SH, Lee TH, Lee S-W, Cho M-H. Biochemical characterization of the rice cinnamyl alcohol dehydrogenase gene family. Molecules. 2018;23(10):2659. doi: 10.3390/molecules23102659 30332817 PMC6222663

[87] Saballos A, Ejeta G, Sanchez E, Kang C, Vermerris W. A genomewide analysis of the cinnamyl alcohol dehydrogenase family in sorghum [*Sorghum bicolor* (L.) Moench] identifies SbCAD2 as the brown midrib6 gene. Genetics. 2009;181(2):783–95. doi: 10.1534/genetics.108.098996 19087955 PMC2644965

[88] Rieseberg TP, Dadras A, Fürst-Jansen JMR, Dhabalia Ashok A, Darienko T, de Vries S, et al. Crossroads in the evolution of plant specialized metabolism. Semin Cell Dev Biol. 2023;134:37–58. doi: 10.1016/j.semcdb.2022.03.004 35292191

[89] de Vries J, de Vries S, Curtis BA, Zhou H, Penny S, Feussner K, et al. Heat stress response in the closest algal relatives of land plants reveals conserved stress signaling circuits. Plant J. 2020;103(3):1025–48. doi: 10.1111/tpj.14782 32333477

[90] Dadras A, Fürst-Jansen JMR, Darienko T, Krone D, Scholz P, Sun S, et al. Environmental gradients reveal stress hubs pre-dating plant terrestrialization. Nat Plants. 2023;9(9):1419–38. doi: 10.1038/s41477-023-01491-0 37640935 PMC10505561

[91] von Schwartzenberg K, Lindner A-C, Gruhn N, Šimura J, Novák O, Strnad M, et al. CHASE domain-containing receptors play an essential role in the cytokinin response of the moss *Physcomitrella patens*. J Exp Bot. 2016;67(3):667–79. doi: 10.1093/jxb/erv479 26596764 PMC4737067

[92] Kieber JJ, Schaller GE. Cytokinin signaling in plant development. Development. 2018;145(4):dev149344. doi: 10.1242/dev.149344 29487105

[93] Mähönen AP, Bonke M, Kauppinen L, Riikonen M, Benfey PN, Helariutta Y. A novel two-component hybrid molecule regulates vascular morphogenesis of the *Arabidopsis* root. Genes Dev. 2000;14(23):2938–43. doi: 10.1101/gad.189200 11114883 PMC317089

[94] Tran L-SP, Urao T, Qin F, Maruyama K, Kakimoto T, Shinozaki K, et al. Functional analysis of AHK1/ATHK1 and cytokinin receptor histidine kinases in response to abscisic acid, drought, and salt stress in *Arabidopsis*. Proc Natl Acad Sci U S A. 2007;104(51):20623–8. doi: 10.1073/pnas.0706547105 18077346 PMC2154481

[95] Dadras A, Duminil P, de Vries S, Irisarri I, Feussner I, de Vries J. Algal origins of core land plant stress response sub-networks. 2025. 10.1111/tpj.70291 PMC12185132 40550109

[96] Rieseberg TP, Dadras A, Darienko T, Post S, Herrfurth C, Fürst-Jansen JMR, et al. Time-resolved oxidative signal convergence across the algae-embryophyte divide. Nat Commun. 2025;16(1):1780. doi: 10.1038/s41467-025-56939-y 39971942 PMC11840003

[97] Kunz CF, Abreu IN, Darienko T, Fürst-Jansen J, Feussner K, Feussner I, et al. Chemodiverse cell system responses to UV in an algal sister of land plants. Curr Biol. 2026;36(10):2673-2687.e6. doi: 10.1016/j.cub.2026.04.015 42092354

[98] Kabbara S, Hérivaux A, Dugé de Bernonville T, Courdavault V, Clastre M, Gastebois A, et al. Diversity and evolution of sensor histidine kinases in eukaryotes. Genome Biol Evol. 2019;11(1):86–108. doi: 10.1093/gbe/evy213 30252070 PMC6324907

[99] Heyl A, Wulfetange K, Pils B, Nielsen N, Romanov GA, Schmülling T. Evolutionary proteomics identifies amino acids essential for ligand-binding of the cytokinin receptor CHASE domain. BMC Evol Biol. 2007;7:62. doi: 10.1186/1471-2148-7-62 17439640 PMC1863423

[100] Stolz A, Riefler M, Lomin SN, Achazi K, Romanov GA, Schmülling T. The specificity of cytokinin signalling in *Arabidopsis thaliana* is mediated by differing ligand affinities and expression profiles of the receptors. Plant J. 2011;67(1):157–68. doi: 10.1111/j.1365-313X.2011.04584.x 21426428

[101] Vogt JHM, Schippers JHM. Setting the PAS, the role of circadian PAS domain proteins during environmental adaptation in plants. Front Plant Sci. 2015;6:513. doi: 10.3389/fpls.2015.00513 26217364 PMC4496561

[102] Ryo M, Yamashino T, Nomoto Y, Goto Y, Ichinose M, Sato K, et al. Light-regulated PAS-containing histidine kinases delay gametophore formation in the moss *Physcomitrella patens*. J Exp Bot. 2018;69(20):4839–51. doi: 10.1093/jxb/ery257 29992239 PMC6137987

[103] Remias D, Procházková L. Ecophysiology of the conjugating green algae (Zygnematophyceae) with emphasis on semiterrestrial habitats and specialized metabolic compounds. Protoplasma. 2026:10.1007/s00709-026-02204-y. doi: 10.1007/s00709-026-02204-y 42062569 PMC7619713

[104] Buschmann H, Zachgo S. The evolution of cell division: From streptophyte algae to land plants. Trends Plant Sci. 2016;21(10):872–83. doi: 10.1016/j.tplants.2016.07.004 27477927

[105] Hall JD, McCourt RM, Delwiche CF. Patterns of cell division in the filamentous Desmidiaceae, close green algal relatives of land plants. Am J Bot. 2008;95(6):643–54. doi: 10.3732/ajb.2007210 21632389

[106] Zegers JMS, Pfeifer L, Darienko T, Schmitt K, Dorsch CA, Kunz CF, et al. Systems acclimation to osmotic stress in zygnematophyte cells. Nat Commun. 2026;17(1):755. doi: 10.1038/s41467-026-68329-z 41545391 PMC12820192

[107] Amborella Genome Project. The Amborella genome and the evolution of flowering plants. Science. 2013;342(6165):1241089. doi: 10.1126/science.1241089 24357323

[108] Li F-W, Nishiyama T, Waller M, Frangedakis E, Keller J, Li Z, et al. Anthoceros genomes illuminate the origin of land plants and the unique biology of hornworts. Nat Plants. 2020;6(3):259–72. doi: 10.1038/s41477-020-0618-2 32170292 PMC8075897

[109] Lamesch P, Berardini TZ, Li D, Swarbreck D, Wilks C, Sasidharan R, et al. The Arabidopsis Information Resource (TAIR): Improved gene annotation and new tools. Nucleic Acids Res. 2012;40(Database issue):D1202-10. doi: 10.1093/nar/gkr1090 22140109 PMC3245047

[110] Hu TT, Pattyn P, Bakker EG, Cao J, Cheng J-F, Clark RM, et al. The *Arabidopsis lyrata* genome sequence and the basis of rapid genome size change. Nat Genet. 2011;43(5):476–81. doi: 10.1038/ng.807 21478890 PMC3083492

[111] Li F-W, Brouwer P, Carretero-Paulet L, Cheng S, de Vries J, Delaux P-M, et al. Fern genomes elucidate land plant evolution and cyanobacterial symbioses. Nat Plants. 2018;4(7):460–72. doi: 10.1038/s41477-018-0188-8 29967517 PMC6786969

[112] Carpenter EJ, Matasci N, Ayyampalayam S, Wu S, Sun J, Yu J, et al. Access to RNA-sequencing data from 1,173 plant species: The 1000 Plant transcriptomes initiative (1KP). Gigascience. 2019;8(10):giz126. doi: 10.1093/gigascience/giz126 31644802 PMC6808545

[113] Moreau H, Verhelst B, Couloux A, Derelle E, Rombauts S, Grimsley N, et al. Gene functionalities and genome structure in *Bathycoccus prasinos* reflect cellular specializations at the base of the green lineage. Genome Biol. 2012;13(8):R74. doi: 10.1186/gb-2012-13-8-r74 22925495 PMC3491373

[114] International Brachypodium Initiative. Genome sequencing and analysis of the model grass *Brachypodium distachyon*. Nature. 2010;463(7282):763–8. doi: 10.1038/nature08747 20148030

[115] Liu S, Liu Y, Yang X, Tong C, Edwards D, Parkin IAP, et al. The *Brassica oleracea* genome reveals the asymmetrical evolution of polyploid genomes. Nat Commun. 2014;5:3930. doi: 10.1038/ncomms4930 24852848 PMC4279128

[116] Goodstein DM, Shu S, Howson R, Neupane R, Hayes RD, Fazo J, et al. Phytozome: A comparative platform for green plant genomics. Nucleic Acids Res. 2012;40(Database issue):D1178-86. doi: 10.1093/nar/gkr944 22110026 PMC3245001

[117] Slotte T, Hazzouri KM, Ågren JA, Koenig D, Maumus F, Guo Y-L, et al. The *Capsella rubella* genome and the genomic consequences of rapid mating system evolution. Nat Genet. 2013;45(7):831–5. doi: 10.1038/ng.2669 23749190

[118] Ming R, Hou S, Feng Y, Yu Q, Dionne-Laporte A, Saw JH, et al. The draft genome of the transgenic tropical fruit tree papaya (*Carica papaya* Linnaeus). Nature. 2008;452(7190):991–6. doi: 10.1038/nature06856 18432245 PMC2836516

[119] Marchant DB, Chen G, Cai S, Chen F, Schafran P, Jenkins J, et al. Dynamic genome evolution in a model fern. Nat Plants. 2022;8(9):1038–51. doi: 10.1038/s41477-022-01226-7 36050461 PMC9477723

[120] Delwiche CF, Cooper E. Green algal transcriptomes for phylogenetics and comparative genomics. 2016. Available from: https://figshare.com/articles/Green_algal_transcriptomes_for_phylogenetics_and_comparative_genomics/120

[121] Merchant SS, Prochnik SE, Vallon O, Harris EH, Karpowicz SJ, Witman GB, et al. The Chlamydomonas genome reveals the evolution of key animal and plant functions. Science. 2007;318(5848):245–50. doi: 10.1126/science.1143609 17932292 PMC2875087

[122] Blaby IK, Blaby-Haas CE, Tourasse N, Hom EFY, Lopez D, Aksoy M, et al. The Chlamydomonas genome project: A decade on. Trends Plant Sci. 2014;19(10):672–80. doi: 10.1016/j.tplants.2014.05.008 24950814 PMC4185214

[123] Cecchin M, Marcolungo L, Rossato M, Girolomoni L, Cosentino E, Cuine S, et al. Chlorella vulgaris genome assembly and annotation reveals the molecular basis for metabolic acclimation to high light conditions. Plant J. 2019;100(6):1289–305. doi: 10.1111/tpj.14508 31437318 PMC6972661

[124] Irisarri I, Darienko T, Pröschold T, Fürst-Jansen JMR, Jamy M, de Vries J. Unexpected cryptic species among streptophyte algae most distant to land plants. Proc Biol Sci. 2021;288(1963):20212168. doi: 10.1098/rspb.2021.2168 34814752 PMC8611356

[125] Wang S, Li L, Li H, Sahu SK, Wang H, Xu Y, et al. Genomes of early-diverging streptophyte algae shed light on plant terrestrialization. Nat Plants. 2020;6(2):95–106. doi: 10.1038/s41477-019-0560-3 31844283 PMC7027972

[126] Kawaguchi YW, Tsuchikane Y, Tanaka K, Taji T, Suzuki Y, Toyoda A, et al. Extensive copy number variation explains genome size variation in the unicellular Zygnematophycean alga, Closterium peracerosum-strigosum-littorale Complex. Genome Biol Evol. 2023;15(8):evad115. doi: 10.1093/gbe/evad115 37348049 PMC10407611

[127] Blanc G, Agarkova I, Grimwood J, Kuo A, Brueggeman A, Dunigan DD, et al. The genome of the polar eukaryotic microalga *Coccomyxa subellipsoidea* reveals traits of cold adaptation. Genome Biol. 2012;13(5):R39. doi: 10.1186/gb-2012-13-5-r39 22630137 PMC3446292

[128] Mundt F, Hanelt D, Harms L, Heinrich S. Darkness-induced effects on gene expression in *Cosmarium crenatum* (Zygnematophyceae) from a polar habitat. Sci Rep. 2019;9(1):10559. doi: 10.1038/s41598-019-47041-7 31332253 PMC6646379

[129] Jackrel SL, Narwani A, Bentlage B, Levine RB, Hietala DC, Savage PE. Ecological engineering helps maximize function in algal oil production. Appl Environ Microbiol. 2018;84(15). doi: 10.1128/AEM.00953-18 29776927 PMC6052273

[130] Wan T, Liu Z-M, Li L-F, Leitch AR, Leitch IJ, Lohaus R, et al. A genome for gnetophytes and early evolution of seed plants. Nat Plants. 2018;4(2):82–9. doi: 10.1038/s41477-017-0097-2 29379155

[131] Su D, Yang L, Shi X, Ma X, Zhou X, Hedges SB, et al. Large-scale phylogenomic analyses reveal the monophyly of bryophytes and neoproterozoic origin of land plants. Mol Biol Evol. 2021;38(8):3332–44. doi: 10.1093/molbev/msab106 33871608 PMC8321542

[132] Li F, Fan G, Lu C, Xiao G, Zou C, Kohel RJ, et al. Genome sequence of cultivated Upland cotton (*Gossypium hirsutum* TM-1) provides insights into genome evolution. Nat Biotechnol. 2015;33(5):524–30. Epub 20150420. doi: 10.1038/nbt.3208 25893780

[133] Chen ZJ, Sreedasyam A, Ando A, Song Q, De Santiago LM, Hulse-Kemp AM, et al. Genomic diversifications of five *Gossypium* allopolyploid species and their impact on cotton improvement. Nat Genet. 2020;52(5):525–33. doi: 10.1038/s41588-020-0614-5 32313247 PMC7203012

[134] Wickell D, Kuo L-Y, Yang H-P, Dhabalia Ashok A, Irisarri I, Dadras A, et al. Underwater CAM photosynthesis elucidated by Isoetes genome. Nat Commun. 2021;12(1):6348. doi: 10.1038/s41467-021-26644-7 34732722 PMC8566536

[135] Hori K, Maruyama F, Fujisawa T, Togashi T, Yamamoto N, Seo M, et al. Klebsormidium flaccidum genome reveals primary factors for plant terrestrial adaptation. Nat Commun. 2014;5:3978. doi: 10.1038/ncomms4978 24865297 PMC4052687

[136] Bowman JL, Kohchi T, Yamato KT, Jenkins J, Shu S, Ishizaki K, et al. Insights into land plant evolution garnered from the *Marchantia polymorpha* genome. Cell. 2017;171(2):287-304.e15. doi: 10.1016/j.cell.2017.09.030 28985561

[137] Rieseberg TP, Dadras A, Bergschmidt LIN, Bierenbroodspot MJ, Fürst-Jansen JMR, Irisarri I, et al. Divergent responses in desiccation experiments in two ecophysiologically different Zygnematophyceae. Physiol Plant. 2023;175(6):e14056. doi: 10.1111/ppl.14056 38148198

[138] Worden AZ, Lee J-H, Mock T, Rouzé P, Simmons MP, Aerts AL, et al. Green evolution and dynamic adaptations revealed by genomes of the marine picoeukaryotes Micromonas. Science. 2009;324(5924):268–72. doi: 10.1126/science.1167222 19359590

[139] Fürst-Jansen JMR, de Vries S, Lorenz M, von Schwartzenberg K, Archibald JM, de Vries J. Submergence of the filamentous Zygnematophyceae Mougeotia induces differential gene expression patterns associated with core metabolism and photosynthesis. Protoplasma. 2022;259(5):1157–74. doi: 10.1007/s00709-021-01730-1 34939169 PMC9385824

[140] Sierro N, Battey JND, Ouadi S, Bakaher N, Bovet L, Willig A, et al. The tobacco genome sequence and its comparison with those of tomato and potato. Nat Commun. 2014;5:3833. doi: 10.1038/ncomms4833 24807620 PMC4024737

[141] Ju C, Van de Poel B, Cooper ED, Thierer JH, Gibbons TR, Delwiche CF, et al. Conservation of ethylene as a plant hormone over 450 million years of evolution. Nat Plants. 2015;1:14004. doi: 10.1038/nplants.2014.4 27246051

[142] Ouyang S, Zhu W, Hamilton J, Lin H, Campbell M, Childs K, et al. The TIGR Rice Genome Annotation Resource: Improvements and new features. Nucleic Acids Res. 2007;35(Database issue):D883-7. doi: 10.1093/nar/gkl976 17145706 PMC1751532

[143] Palenik B, Grimwood J, Aerts A, Rouzé P, Salamov A, Putnam N, et al. The tiny eukaryote *Ostreococcus* provides genomic insights into the paradox of plankton speciation. Proc Natl Acad Sci U S A. 2007;104(18):7705–10. doi: 10.1073/pnas.0611046104 17460045 PMC1863510

[144] Lang D, Ullrich KK, Murat F, Fuchs J, Jenkins J, Haas FB, et al. The *Physcomitrella* patens chromosome-scale assembly reveals moss genome structure and evolution. Plant J. 2018;93(3):515–33. doi: 10.1111/tpj.13801 29237241

[145] Bi G, Zhao S, Yao J, Wang H, Zhao M, Sun Y, et al. Near telomere-to-telomere genome of the model plant *Physcomitrium* patens. Nat Plants. 2024;10(2):327–43. doi: 10.1038/s41477-023-01614-7 38278953

[146] Nystedt B, Street NR, Wetterbom A, Zuccolo A, Lin Y-C, Scofield DG, et al. The Norway spruce genome sequence and conifer genome evolution. Nature. 2013;497(7451):579–84. doi: 10.1038/nature12211 23698360

[147] Banks JA, Nishiyama T, Hasebe M, Bowman JL, Gribskov M, dePamphilis C, et al. The *Selaginella* genome identifies genetic changes associated with the evolution of vascular plants. Science. 2011;332(6032):960–3. doi: 10.1126/science.1203810 21551031 PMC3166216

[148] Tomato Genome Consortium. The tomato genome sequence provides insights into fleshy fruit evolution. Nature. 2012;485(7400):635–41. doi: 10.1038/nature11119 22660326 PMC3378239

[149] Healey AL, Piatkowski B, Lovell JT, Sreedasyam A, Carey SB, Mamidi S, et al. Newly identified sex chromosomes in the *Sphagnum* (peat moss) genome alter carbon sequestration and ecosystem dynamics. Nat Plants. 2023;9(2):238–54. doi: 10.1038/s41477-022-01333-5 36747050 PMC9946827

[150] Paul BG, Burstein D, Castelle CJ, Handa S, Arambula D, Czornyj E, et al. Retroelement-guided protein diversification abounds in vast lineages of Bacteria and Archaea. Nat Microbiol. 2017;2:17045. doi: 10.1038/nmicrobiol.2017.45 28368387 PMC5436926

[151] Argout X, Salse J, Aury J-M, Guiltinan MJ, Droc G, Gouzy J, et al. The genome of *Theobroma cacao*. Nat Genet. 2011;43(2):101–8. doi: 10.1038/ng.736 21186351

[152] Motamayor JC, Mockaitis K, Schmutz J, Haiminen N, Livingstone D 3rd, Cornejo O, et al. The genome sequence of the most widely cultivated cacao type and its use to identify candidate genes regulating pod color. Genome Biol. 2013;14(6):r53. doi: 10.1186/gb-2013-14-6-r53 23731509 PMC4053823

[153] International Wheat Genome Sequencing Consortium (IWGSC). Shifting the limits in wheat research and breeding using a fully annotated reference genome. Science. 2018;361(6403):eaar7191. doi: 10.1126/science.aar7191 30115783

[154] De Clerck O, Kao S-M, Bogaert KA, Blomme J, Foflonker F, Kwantes M, et al. Insights into the evolution of multicellularity from the sea lettuce genome. Curr Biol. 2018;28(18):2921-2933.e5. doi: 10.1016/j.cub.2018.08.015 30220504

[155] Prochnik SE, Umen J, Nedelcu AM, Hallmann A, Miller SM, Nishii I, et al. Genomic analysis of organismal complexity in the multicellular green alga *Volvox carteri*. Science. 2010;329(5988):223–6. doi: 10.1126/science.1188800 20616280 PMC2993248

[156] Hess S, Eme L, Roger AJ, Simpson AGB. A natural toroidal microswimmer with a rotary eukaryotic flagellum. Nat Microbiol. 2019;4(10):1620–6. doi: 10.1038/s41564-019-0478-6 31182800

[157] Rippin M, Pichrtová M, Arc E, Kranner I, Becker B, Holzinger A. Metatranscriptomic and metabolite profiling reveals vertical heterogeneity within a *Zygnema* green algal mat from Svalbard (High Arctic). Environ Microbiol. 2019;21(11):4283–99. doi: 10.1111/1462-2920.14788 31454446 PMC6899726

[158] Seppey M, Manni M, Zdobnov EM. BUSCO: Assessing genome assembly and annotation completeness. Methods Mol Biol. 2019;1962:227–45. doi: 10.1007/978-1-4939-9173-0_14 31020564

[159] Steenwyk JL, Buida TJ 3rd, Li Y, Shen X-X, Rokas A. ClipKIT: A multiple sequence alignment trimming software for accurate phylogenomic inference. PLoS Biol. 2020;18(12):e3001007. doi: 10.1371/journal.pbio.3001007 33264284 PMC7735675

[160] Jones P, Binns D, Chang H-Y, Fraser M, Li W, McAnulla C, et al. InterProScan 5: Genome-scale protein function classification. Bioinformatics. 2014;30(9):1236–40. doi: 10.1093/bioinformatics/btu031 24451626 PMC3998142

[161] Nguyen L-T, Schmidt HA, von Haeseler A, Minh BQ. IQ-TREE: A fast and effective stochastic algorithm for estimating maximum-likelihood phylogenies. Mol Biol Evol. 2015;32(1):268–74. doi: 10.1093/molbev/msu300 25371430 PMC4271533

[162] Minh BQ, Schmidt HA, Chernomor O, Schrempf D, Woodhams MD, von Haeseler A, et al. IQ-TREE 2: New models and efficient methods for phylogenetic inference in the genomic era. Mol Biol Evol. 2020;37(5):1530–4. doi: 10.1093/molbev/msaa015 32011700 PMC7182206

[163] Wong T, Ly-Trong N, Ren H, Baños H, Roger A, Susko E. IQ-TREE 3: Phylogenomic inference software using complex evolutionary models. EcoEvoRxiv. 2025. doi: 10.32942/x2p62n PMC13191116 42085559

[164] Katoh K, Standley DM. MAFFT multiple sequence alignment software version 7: Improvements in performance and usability. Mol Biol Evol. 2013;30(4):772–80. doi: 10.1093/molbev/mst010 23329690 PMC3603318

[165] Emms DM, Kelly S. OrthoFinder: Solving fundamental biases in whole genome comparisons dramatically improves orthogroup inference accuracy. Genome Biol. 2015;16(1):157. doi: 10.1186/s13059-015-0721-2 26243257 PMC4531804

[166] Emms DM, Kelly S. OrthoFinder: phylogenetic orthology inference for comparative genomics. Genome Biol. 2019;20(1):238. doi: 10.1186/s13059-019-1832-y 31727128 PMC6857279

[167] Yang Z. PAML 4: Phylogenetic analysis by maximum likelihood. Mol Biol Evol. 2007;24(8):1586–91. doi: 10.1093/molbev/msm088 17483113

[168] Revell LJ. phytools: An R package for phylogenetic comparative biology (and other things). Methods Ecol Evol. 2011;3(2):217–23. doi: 10.1111/j.2041-210x.2011.00169.x

[169] Wang H-C, Minh BQ, Susko E, Roger AJ. Modeling site heterogeneity with posterior mean site frequency profiles accelerates accurate phylogenomic estimation. Syst Biol. 2018;67(2):216–35. doi: 10.1093/sysbio/syx068 28950365

[170] Whelan S, Irisarri I, Burki F. PREQUAL: Detecting non-homologous characters in sets of unaligned homologous sequences. Bioinformatics. 2018;34(22):3929–30. doi: 10.1093/bioinformatics/bty448 29868763

[171] Rambaut A, Drummond AJ, Xie D, Baele G, Suchard MA. Posterior summarization in bayesian phylogenetics using tracer 1.7. Syst Biol. 2018;67(5):901–4. doi: 10.1093/sysbio/syy032 29718447 PMC6101584

[172] Capella-Gutiérrez S, Silla-Martínez JM, Gabaldón T. trimAl: A tool for automated alignment trimming in large-scale phylogenetic analyses. Bioinformatics. 2009;25(15):1972–3. doi: 10.1093/bioinformatics/btp348 19505945 PMC2712344

[173] Bolger AM, Lohse M, Usadel B. Trimmomatic: A flexible trimmer for Illumina sequence data. Bioinformatics. 2014;30(15):2114–20. doi: 10.1093/bioinformatics/btu170 24695404 PMC4103590

[174] Friedl T, Lorenz M. The culture collection of algae at Göttingen University (SAG): A biological resource for biotechnological and biodiversity research. Proc Environ Sci. 2012;15:110–7. doi: 10.1016/j.proenv.2012.05.015

[175] Schlösser UC. Additions to the culture collection of algae since 1994. Bot Acta. 1997;110(5):424–9. doi: 10.1111/j.1438-8677.1997.tb00659.x

[176] Schlösser UG. SAG: Sammlung von Algenkulturen at the University of Göttingen catalogue of strains. Bot Acta. 1994;107(3):113–86. doi: 10.1111/j.1438-8677.1994.tb00784.x

[177] Haas BJ, Papanicolaou A, Yassour M, Grabherr M, Blood PD, Bowden J, et al. De novo transcript sequence reconstruction from RNA-seq using the Trinity platform for reference generation and analysis. Nat Protoc. 2013;8(8):1494–512. doi: 10.1038/nprot.2013.084 23845962 PMC3875132

[178] Davidson NM, Hawkins ADK, Oshlack A. SuperTranscripts: A data driven reference for analysis and visualisation of transcriptomes. Genome Biol. 2017;18(1):148. doi: 10.1186/s13059-017-1284-1 28778180 PMC5543425

[179] Steinegger M, Söding J. MMseqs2 enables sensitive protein sequence searching for the analysis of massive data sets. Nat Biotechnol. 2017;35(11):1026–8. doi: 10.1038/nbt.3988 29035372

[180] Brown JW, Walker JF, Smith SA. Phyx: Phylogenetic tools for unix. Bioinformatics. 2017;33(12):1886–8. doi: 10.1093/bioinformatics/btx063 28174903 PMC5870855

[181] Hoang DT, Chernomor O, von Haeseler A, Minh BQ, Vinh LS. UFBoot2: Improving the ultrafast bootstrap approximation. Mol Biol Evol. 2018;35(2):518–22. doi: 10.1093/molbev/msx281 29077904 PMC5850222

[182] Yang Z, Kumar S, Nei M. A new method of inference of ancestral nucleotide and amino acid sequences. Genetics. 1995;141(4):1641–50. doi: 10.1093/genetics/141.4.1641 8601501 PMC1206894

[183] Minh BQ, Nguyen MAT, von Haeseler A. Ultrafast approximation for phylogenetic bootstrap. Mol Biol Evol. 2013;30(5):1188–95. doi: 10.1093/molbev/mst024 23418397 PMC3670741

