# Supplementary material for: A deep genetic structure phylogenomically frames the closest algal relatives of land plants
Source: PLoS Biol. 2026 Nov 3;24(11):e3004005. doi: 10.1371/journal.pbio.3004005 (PMC13730384; doi:10.1371/journal.pbio.3004005)
Supplement: Supplementary file 1 [file pbio.3004005.s001.pdf]

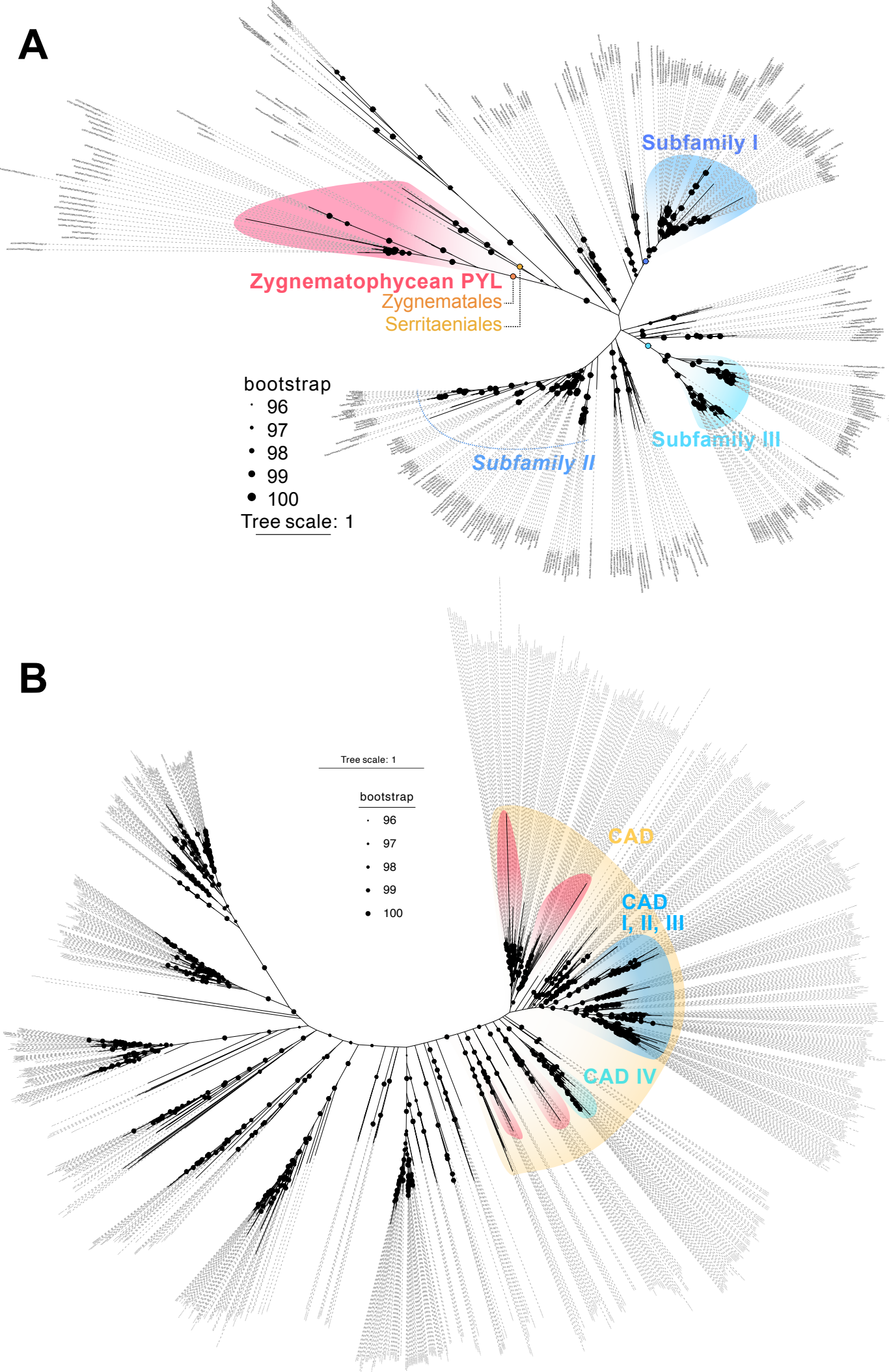

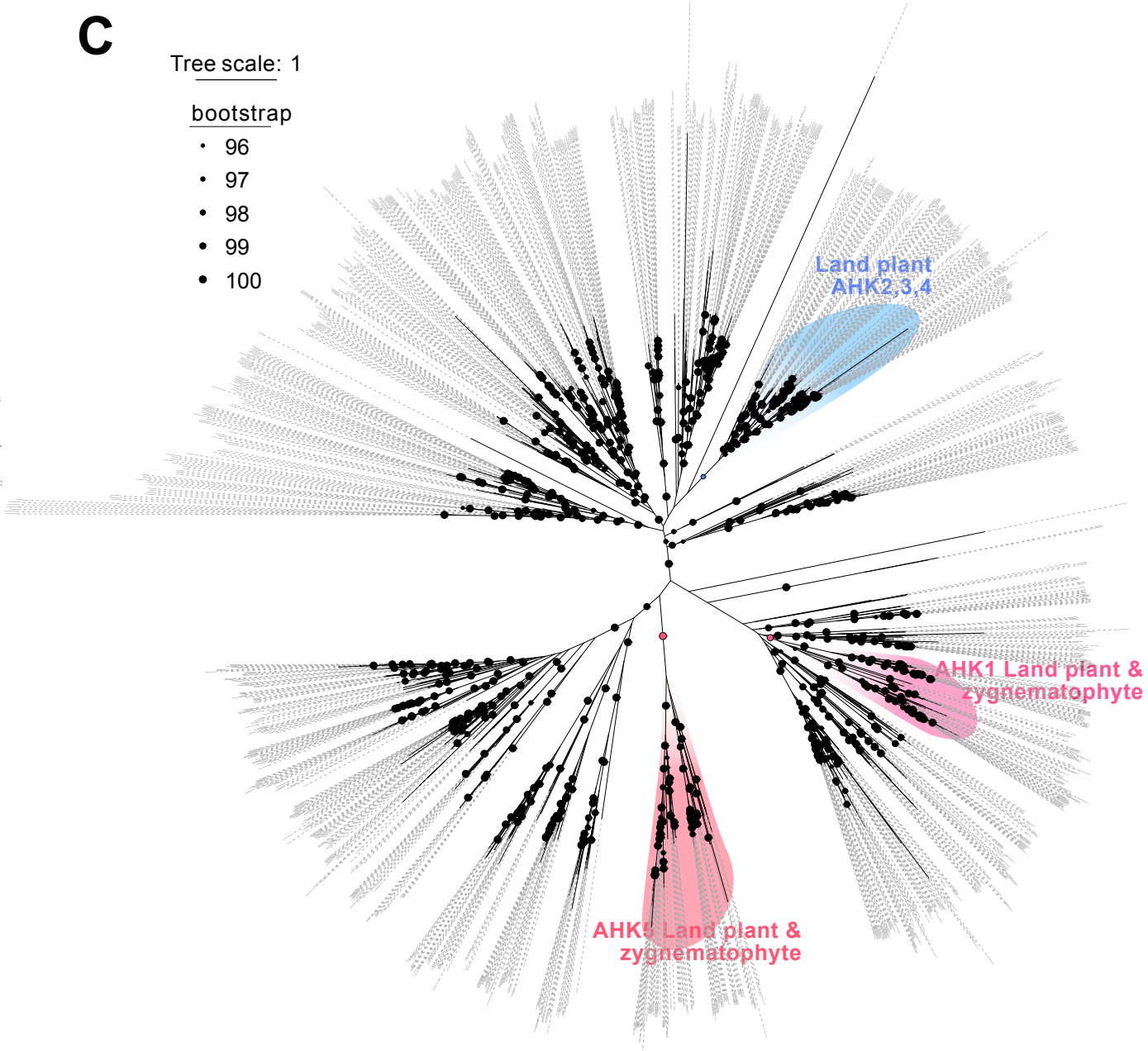

Figure S1. Fully labelled phylogenetic trees of signature gene families. Related to Figure 3. Fully labeled phylogenies shown in Figure 3. The data and code needed to generate this Figure can be found in https://zenodo.org/records/18392035 as well as https://doi.org/10.5281/zenodo.22172294 on Zenodo
