# Supplementary material for: A deep genetic structure phylogenomically frames the closest algal relatives of land plants
Source: PLoS Biol. 2026 Nov 3;24(11):e3004005. doi: 10.1371/journal.pbio.3004005 (PMC13730384; doi:10.1371/journal.pbio.3004005)
Supplement: Supplementary file 2 [file pbio.3004005.s004.docx]

Dear Dr de Vries,

Thank you for your patience while your manuscript "A deep genetic structure phylogenomically frames the closest algal relatives of land plants" was peer-reviewed at PLOS Biology. It has now been evaluated by the PLOS Biology editors, an Academic Editor with relevant expertise, and by several independent reviewers. 

Based on the reviews and on our Academic Editor's assessment of your revision, we are likely to accept this manuscript for publication, provided you satisfactorily address the remaining points raised by the reviewers. Please also make sure to address the following data and other policy-related requests.

IMPORTANT - please attend to the following:

a) Thank you for providing the underlying data and code on GitHub. However, because GitHub repositories can be readily changed or deleted, please make a permanent, DOI assigned copy (e.g. in Zenodo) and provide the corresponding URL.
**>>>>AU: Done: https://doi.org/10.5281/zenodo.22172294**

b) Please make the data deposited on Zenodo (<https://doi.org/10.5281/zenodo.18392035>) publicly available at this stage. For instance, we require the data directly underlying the trees shown in Figs 1A, 2, 3ABC and S1AB (the data required to generate these trees - potentially just a treefile).
**>>>>AU: Done.**


c) Please clearly cite the location of the data in all relevant main and supplementary figure legends, for example: “The data underlying this figure can be found at [doi.org/10.xxx/zenodo.xxxxx](http://doi.org/10.xxx/zenodo.xxxxx).” We understand that this may appear repetitive, but it helps make the figures and their legends more self contained.
**>>>>AU: Done.**

d) Please make the raw data deposited on NCBI Bioproject (PRJNA1416270) publicly available at this stage.

**>>>>AU: Done.**

e) Please include the URLs of your funders in the Financial Disclosure statement.

**>>>>AU: Done.**

f) Reviewer3 in their point 9 suggests improving the title. I am happy  to discuss alternatives by email.

**>>>>AU: We agree that the title is a bit unusual but we have long thought about how to best highlight the finding that was the biggest surprise and has in our view also the highest “newsworthiness”.**

g) Our Academic editor has some comments (below). Please address these presentational comments
**>>>>AU: All comments have been addressed.**

"One minor point is that I find the three main figures to be very geared toward an algal/plant audience. It would be nice if the authors could edit these to be a bit more accessible to a general audience, e.g. 1) making the images of the organisms more directly linked to the specific phylogenetic clades (I am unclear currently which lineages match to which photos), 2) highlight land plants vs. other algae in Figure 2 so the cellular transitions make a bit more sense, and 3) Perhaps add some further annotation or context for Figure 3 so it doesn't come across as so genetics-heavy. I think improving the figures with these minor edits would be better for the broad readership of PLOS Biology."

**>>>>AU: Very good points, well taken. We now have: (1) labeled the micrographs in figure 1 according to the color code we use for the clades; (2) highlighted the land plants in figure 2; and (3) added little symbols to figure 3 to explain the functionality of the different enzymes that were analysed in the respective phylogenetic trees.**

As you address these items, please take this last chance to review your reference list to ensure that it is complete and correct. If you have cited papers that have been retracted, please include the rationale for doing so in the manuscript text, or remove these references and replace them with relevant current references. Any changes to the reference list should be mentioned in the cover letter that accompanies your revised manuscript.

In addition to these revisions, you may need to complete some formatting changes, which you will receive in a follow up email. A member of our team will be in touch with a set of requests shortly. If you do not receive a separate email within a few days, please assume that checks have been completed, and no additional changes are required.

We expect to receive your revised manuscript within two weeks. 

To submit your revision, please go to <https://www.editorialmanager.com/pbiology/> and log in as an Author. Click the link labelled 'Submissions Needing Revision' to find your submission record. Your revised submission must include the following:
-  a cover letter that should detail your responses to any editorial requests, if applicable, and whether changes have been made to the reference list
-  a Response to Reviewers file that provides a detailed response to the reviewers' comments (if applicable, if not applicable please do not delete your existing 'Response to Reviewers' file.)
-  a track-changes file indicating any changes that you have made to the manuscript. 

NOTE: If Supporting Information files are included with your article, note that these are not copyedited and will be published as they are submitted. Please ensure that these files are legible and of high quality (at least 300 dpi) in an easily accessible file format. For this reason, please be aware that any references listed in an SI file will not be indexed. For more information, see our Supporting Information guidelines:
<https://journals.plos.org/plosbiology/s/supporting-information>  

*Published Peer Review History*
Please note that you may have the opportunity to make the peer review history publicly available. The record will include editor decision letters (with reviews) and your responses to reviewer comments. If eligible, we will contact you to opt in or out. Please see here for more details:
<https://plos.org/published-peer-review-history/>

*Press*
Should you, your institution's press office or the journal office choose to press release your paper, please ensure you have opted out of Early Article Posting on the submission form. We ask that you notify us as soon as possible if you or your institution is planning to press release the article.

*Protocols deposition*
To enhance the reproducibility of your results, we recommend that if applicable you deposit your laboratory protocols in [protocols.io](http://protocols.io), where a protocol can be assigned its own identifier (DOI) such that it can be cited independently in the future. Additionally, PLOS ONE offers an option for publishing peer-reviewed Lab Protocol articles, which describe protocols hosted on [protocols.io](http://protocols.io). Read more information on sharing protocols at <https://plos.org/protocols?utm_medium=editorial-email&utm_source=authorletters&utm_campaign=protocols>


Please do not hesitate to contact me should you have any questions.

Sincerely,

Ankiit

Ankiit Ahluwalia, 

[aahluwalia@plos.org](mailto:aahluwalia@plos.org)
PLOS Biology

----------

**>>>Dear Dr Ahluwalia**

**Thank you for handling our manuscript. We are pleased that the reviewers found our work interesting and the manuscript to be well-written.**

**In this revised version of our manuscript, we have made sure to tackle all of the constructive comments made by the reviewers; we have labelled our responses with “>>>>AU:”.**

**Note that in response to reviewer 2, we have added several references to the literature to provide more background information on the gene families that have been studied (Fig. 3).**

**On behalf of all co-authors, I remain**

**Yours sincerely**

**Jan de Vries**

**********************************

------------------------------------------------------------------------
****[DELETE AS APPROPRIATE]****
ETHICS STATEMENT:

-- Please include the full name of the IACUC/ethics committee that reviewed and approved the animal care and use protocol/permit/project license. Please also include an approval number.

-- Please include the specific national or international regulations/guidelines to which your animal care and use protocol adhered. Please note that institutional or accreditation organization guidelines (such as AAALAC) do not meet this requirement.

-- Please include information about the form of consent (written/oral) given for research involving human participants. All research involving human participants must have been approved by the authors' Institutional Review Board (IRB) or an equivalent committee, and must have been conducted according to the principles expressed in the Declaration of Helsinki.

------------------------------------------------------------------------
DATA POLICY:

You may be aware of the PLOS Data Policy, which requires that all data be made available without restriction: <http://journals.plos.org/plosbiology/s/data-availability>. For more information, please also see this editorial: <http://dx.doi.org/10.1371/journal.pbio.1001797>

Note that we do not require all raw data. Rather, we ask that all individual quantitative observations that underlie the data summarized in the figures and results of your paper be made available in one of the following forms:

1) Supplementary files (e.g., excel). Please ensure that all data files are uploaded as 'Supporting Information' and are invariably referred to (in the manuscript, figure legends, and the Description field when uploading your files) using the following format verbatim: S1 Data, S2 Data, etc. Multiple panels of a single or even several figures can be included as multiple sheets in one excel file that is saved using exactly the following convention: S1_Data.xlsx (using an underscore).

2) Deposition in a publicly available repository. Please also provide the accession code or a reviewer link so that we may view your data before publication. 

Regardless of the method selected, please ensure that you provide the individual numerical values that underlie the summary data displayed in the following figure panels as they are essential for readers to assess your analysis and to reproduce it:
[Figs….]
NOTE: the numerical data provided should include all replicates AND the way in which the plotted mean and errors were derived (it should not present only the mean/average values).

Please also ensure that figure legends in your manuscript include information on where the underlying data can be found, and ensure your supplemental data file/s has a legend.

Please ensure that your Data Statement in the submission system accurately describes where your data can be found.

------------------------------------------------------------------------
APPROPRIATE DATA PRESENTATION? 
(If you think the paper is not using best practice, add the following)

- Please ensure that you are using best practice for statistical reporting and data presentation. These are our guidelines <https://journals.plos.org/plosbiology/s/best-practices-in-research-reporting#loc-statistical-reporting> and a useful resource  on data presentation <https://journals.plos.org/plosbiology/article?id=10.1371/journal.pbio.1002128>

- If you are reporting experiments where n ≤ 5, please plot each individual data point.

**>>>>AU: All adhered to.**

------------------------------------------------------------------------

CODE POLICY

Per journal policy, if you have generated any custom code during the course of this investigation, please make it available without restrictions. Please ensure that the code is sufficiently well documented and reusable, and that your Data Statement in the Editorial Manager submission system accurately describes where your code can be found. More information on our Code Policy, what and how to share can be found here: <https://journals.plos.org/plosbiology/s/code-availability>

Please note that we cannot accept sole deposition of code in GitHub, as this could be changed after publication. However, you can archive this version of your publicly available GitHub code to Zenodo. Once you do this, it will generate a DOI number, which you will need to provide in the Data Accessibility Statement (you are welcome to also provide the GitHub access information). See the process for doing this here: <https://docs.github.com/en/repositories/archiving-a-github-repository/referencing-and-citing-content>
**>>>>AU: A comprehensive code and methodological overview is now available both at https://github.com/mjbieren/Zygnematophyceae_Phylogenomics and https://zenodo.org/records/18392035 as well as https://doi.org/10.5281/zenodo.22172294 on Zenodo**

------------------------------------------------------------------------
SPECIES INDICATED IN THE ABSTRACT? 
- Please note that per journal policy, the model system/species studied should be clearly stated in the abstract of your manuscript.  

**EDITOR, CONSIDER ALSO INCLUDING ORGANISM IN THE TITLE, IF SENSICAL/RELEVANT AND/OR TO AVOID BEING MISLEADING/OVERREACHING (not always preferred)**
**>>>>AU: We have worked with 104 strains of dozens of different species from the class of Zygnematophyceae. We clearly state the class we worked with and how many.**
------------------------------------------------------------------------
BLOT AND GEL REPORTING REQUIREMENTS:

We require the original, uncropped and minimally adjusted images supporting all blot and gel results reported in an article's figures or Supporting Information files. We will require these files before a manuscript can be accepted so please prepare and upload them now. Please carefully read our guidelines for how to prepare and upload this data: <https://journals.plos.org/plosbiology/s/figures#loc-blot-and-gel-reporting-requirements>
**>>>>AU: n/a**
------------------------------------------------------------------------
DATA NOT SHOWN?

- Please note that per journal policy, we do not allow the mention of "data not shown", "personal communication", "manuscript in preparation" or other references to data that is not publicly available or contained within this manuscript. Please either remove mention of these data or provide figures presenting the results and the data underlying the figure(s).
**>>>>AU: n/a**
------------------------------------------------------------------------

Reviewer remarks:


Reviewer #1: A deep genetic structure phylogenomically frames the closest algal relatives of land Plants
Bierenbroodspot et al

This manuscript presents a comprehensive phylogenomic analysis of streptophyte algae to clarify the evolutionary origin of land plants. Using a genome-scale dataset, the authors resolve the deep evolutionary relationships within streptophyte algae and provide new views on the relationships among the closest algal relatives of embryophytes. I think the study significantly advances our understanding of the evolutionary transition from aquatic algae to terrestrial plants and provides a framework for investigating the origin of key land plant traits.
Overall, I consider this to be a high-quality study with the potential for substantial impact. It is likely to become an important reference for research on early streptophyte evolution and the evolutionary context of land plant origins. I found the analyses rigorous, and I do not have any major concerns regarding the study design. The remaining uncertainties are largely those inherent to deep phylogenomic reconstruction rather than shortcomings of the work itself.

**>>>>AU: Thank you for the kind words, the appreciation of our work, and the helpful comments to improve our manuscript.**

I have a few major comments:
1) Ancestral reconstruction of morphological traits: one potential caveat concerns the reconstruction of morphological characters. I believe many streptophyte algae have complex life cycles, with different generations or life stages exhibiting distinct morphologies. If this is the case for some of the taxa included here, trait coding based on a single life stage could bias ancestral state reconstruction. I appreciate that complete life cycle information may not be available for many species, but I think this limitation should be explicitly acknowledged and discussed.

**>>>>AU: There is indeed very little influence of the different life cycle morphologies on our analyses, as the life cycle of most streptophyte algae is dominated by the vegetative stage, which is also the one with the most elaborate body plan. To make all of this clearer we now state “For ACSR, we considered the morphological capabilities of all organisms when coding the characters, although these characters primarily reflect the capabilities of the vegetative cells. In Zygnematophyceae in particular, the life cycle does not influence their overall growth capabilities, as it comprises only vegetative cells and zygospores”.**

2) Definition of genomic streamlining: it is not entirely clear how the trait "genomic streamlining" was defined and quantified. I recommend providing additional methodological details describing the criteria used to assign this trait.

**>>>>AU: This was not quantified in this study—to make this clearer, we now state explicitly “described in Goldbecker et al. [13]”**

3) Interpretation of PYL loss:the discussion of PYL loss should be interpreted more cautiously. Demonstrating true gene loss can be challenging, particularly when genome assemblies are incomplete or when transcriptome-based data are used, where the absence of expression does not necessarily imply gene absence. It would be helpful if the authors discussed these limitations and moderated the statement that PYL loss "underpins the idea that extensive rewiring in the homologous chassis occurred."

**>>>>AU: We have added the statement “confirming this absence will require broader taxonomic sampling, supported by contiguous genome assemblies rather than relying largely on transcriptomic data.” and toned down the statement on PYL loss-necessitated rewiring.**

4) Description of morphological traits: since ancestral reconstruction of morphological traits is a central component of the study, I think the manuscript would benefit from providing clearer definitions of the characters used. For example, I was not entirely sure what was meant by a "chain-like filament," and I felt I should not have to consult reference 97 to understand such an important character. Similarly, terms such as "desmid cell" may not be familiar to a broad readership. Brief definitions or illustrations within the manuscript would make the work more accessible.

**>>>>AU: Point well taken. We have added early on “The cells of these Desmidiales, characteristic of a desmid cell, are typically unicellular and composed of two symmetrical semicells joined at a central isthmus. Each semicell generally contains a chloroplast, and the cells exhibit diverse shapes, ranging from simple elongate or spherical forms to highly ornamented, lobed, or deeply constricted morphologies.” And later when we explain the chain like desmids “Chain-like filaments of desmid cells consist of individual, typically unicellular desmid cells arranged end-to-end, forming elongated, unbranched chains. Adjacent cells remain connected following cell division, while retaining their characteristic desmid morphology, often with distinct semicells separated by a median constriction.”**

5) Evolution of multicellularity and body plans. The manuscript discusses the independent evolution of filamentous growth several times, but I found it equally interesting that the "macrophyte" body plan also appears to have evolved independently on multiple occasions. Moreover, unless I have misunderstood the reconstruction, these transitions seem to occur directly from unicellular ancestors rather than through filamentous intermediates. If this interpretation is correct, I think it deserves further discussion, as it has potentially interesting implications for the evolution of complex multicellularity in streptophyte algae.

**>>>>AU: We fully agree that this is a very interesting topic. However the taxon sampling here was focused on the zygnematophytes. We would therefore refrain from making any conclusions about the other lineages. This entire topic was recently discussed by us, which we now highlight early on stating “for a recent review on the multiple evolutionary emergences of (sometimes very complex) multicellular streptophyte bodies, see Darienko et al. [42].”**

6) Evolutionary models for trait transitions. I also wondered whether the authors considered using phyllogenetic comparative methods to estimate transition probabilities among traits. Such approaches might provide complementary insights into the evolutionary dynamics of these characters. If the authors considered these analyses but chose not to pursue them, it would be useful to briefly explain why.

**>>>>AU: This is indeed what has been conducted. ACSR is a phylogenetic comparative method that estimates the probability of character/trait states at ancestral nodes given the observed states at the tips, the phylogeny, and an assumed model of trait evolution. So it takes the branch lengths into consideration and the transition rates.**

Minor
Please add scale bar to photos in Figure 1
**>>>>AU: Done.**


Reviewer #2: In this study, Bierenbroodspot and coauthors provide 43 novel transcriptomes and phylogenomic analyses within the class Zygnematophyceae. 
This class of algae is the closest streptophyte algal relative of embryophytes.
Beyond the detailed phylogeny of this class, this study also addresses more general issues such as the evolution of multicellularity and the evolution of gene families associated with the ecological success of land plants.
The authors completed the available sequence data with 43 transcriptomes from approximately 36 species, adding up to 104 zygnematophycean algae, nine other streptophyte algae, eight land plants, and four chlorophyte algae as an outgroup.
The authors first confirm the phylogenetic position of the Zygnematophyceae class as the closest streptophyte algal relatives of embryophytes (Figure 1).
They use ancestral state reconstruction to infer the ancestral morphology (unicellular/ filament/ sarcinoid) of this class. The analyses suggest the ancestral state is unicellular and that there have been seven independent transitions to a filamentous body plan (Figure 2).
They then investigate a few families : the abscisic acid receptor gene, and gene families from the phenylpropanoid pathway (Figure 3).
The manuscript is very well written, synthetic, the analyses and results are sound, and I have only minor comments : I think providing additional context and information would further increase its readability. Below are some suggestions in this direction.

**>>>>AU: Thank you for the kind words and the appreciation of our work.**

1.	As a non-Zygnematophyceae expert, I believe this will be the case for many readers, I would have appreciated some more general context about previous quests of the gene families involved in terrestralisation (e.g. <https://doi.org/10.1371/journal.pbio.3002608>), as well as the ancestry of the genes and gene families the authors target in this study. Could you provide more information about the presence/absence of homologs in more ancestral lineages, before describing the independent gene family expansions in Zygnematophyceae and Embryophytes ? May the annotation of the black branches in Figure 3 provide this information ?

**>>>>AU: Thank you for this remark. We have added more introductory content.**

2.	Methods section : line 141 Could you clarify why you excluded clades when only one protein contained the domain, Couldn't it be that there is juste one copy of this gene in the clade ? 
**>>>>AU: We agree that, in principle, a single protein containing the domain could represent the sole copy of the corresponding gene within a clade. However, we excluded such cases because a domain detected in only a single protein provides limited evidence for a clade-level inference and is particularly susceptible to sporadic annotation errors, gene-model artefacts, or other sequence-specific false positives. More importantly, an observation based on a single protein (n = 1) provides very limited support for drawing conclusions about the broader evolutionary pattern within that clade. We therefore adopted a conservative filtering criterion and retained only labels for clades in which the domain was detected in more than one protein, thereby reducing the influence of potentially spurious individual observations on the overall analysis. We have clarified this rationale in the revised Methods section.**


Reviewer #3: This manuscript presents a major phylogenomic investigation of the Zygnematophyceae, the closest algal relatives of land plants. By generating 43 new transcriptomes and integrating them with publicly available datasets, the authors establish an extensive phylogenomic framework comprising 104 zygnematophyte taxa and 2,243 loci. The resulting dataset considerably expands taxonomic representation across the group and provides an important resource for future studies of streptophyte algal evolution and plant terrestrialization.
Overall, I found the manuscript to be well written, the analytical framework carefully designed, and the conclusions largely supported by the data. In particular, the breadth of taxon sampling represents a significant advance over previous studies and allows the authors to address several evolutionary questions within a much stronger phylogenetic context than has previously been possible. The paper will be of broad interest to researchers working on plant evolution, algal diversity, and the origins of land plant traits.

**>>>>AU: Thank you for the kind words and the appreciation of our work.**

I have only a few suggestions that I believe would further strengthen the manuscript.

**>>>>AU: Thank you. We have used all your very helpful comments to improve our manuscript.**

COMMENTS

1. Figure 1. I recommend indicating in panel A which taxa were newly sampled in this study. In addition, some labels and clade names could be enlarged to improve readability.

**>>>>AU: Good point. Done.**

2. Lines 136-142. The discussion of the exceptionally long branch leading to extant Spirogyrales is thought-provoking. However, some of the interpretations presented, such as extensive extinction or the existence of large amounts of unsampled diversity, are necessarily speculative. I suggest presenting these possibilities more explicitly as alternative hypotheses rather than preferred explanations. Additional explanations for long branches include accelerated rates of molecular evolution, heterogeneous selection pressures, compositional biases, and transcriptome-derived biases.

**>>>>AU: Point well taken. We have expanded the discussion in this section.**

3. Lines 92-96 and 515-518. The ancestral character state reconstruction constitutes an important aspect of the manuscript, particularly the inference of repeated origins of filamentous growth and a unicellular ancestor of Zygnematophyceae. It would be helpful if the authors could provide additional methodological details, as the description in the Methods section is currently quite brief and may make the analysis difficult to reproduce. More detailed information may be available on the authors' Zenodo and GitHub sites, but I was unable to access them. A brief discussion of the assumptions underlying the reconstruction model would also be useful.

**>>>>AU: We have added details on how to interpret the ACSR and the coding.**

4. Figure 2 and legend. Additional details regarding the output of the ancestral character state reconstruction would help readers interpret the results. In particular, how should circles containing multiple-colored sectors be interpreted? For example, what is the inferred likelihood of different body plans for the common ancestor of Zygnematophyceae and land plants? Was this ancestor more likely to have been unicellular than multicellular? Uncertainty in reconstructed states should be shown if possible.

**>>>>AU: Good point. We added an explanation that states “Our data bolster the concept that the LCA of Zygnematophyceae was an ancestral unicell (Figure 2), which our analyses recover with a posterior probability of 0.988 (the body plan of the LCA of land plants and Zygneamtophyceae could not be robustly inferred)”. We have further added a statement on how to read figure 2 and how to interpret the posterior probabilities.**

5. Lines 209-211. The authors state that "both in the case of the Charophyceae and the discoidal Coleochaete species, the complexity of body plans is likely the result of secondary gains." This raises the question of whether the common ancestor of Charophyceae and Coleochaetophyceae was also unicellular, as inferred for the common ancestor of Zygnematophyceae. Additional discussion of this point would be helpful.

**>>>>AU: We fully agree that this is a very interesting topic. However the taxon sampling here was focused on the zygnematophytes. We would therefore refrain from making any conclusions about the other lineages. This entire topic was recently discussed by us, which we now highlight early on stating “for a recent review on the multiple evolutionary emergences of (sometimes very complex) multicellular streptophyte bodies, see Darienko et al. [42].”**

6. Lines 229-232. The analyses of PYL homolog distribution across Zygnematophyceae are interesting and provide valuable insights into the evolutionary history of this gene family. However, because much of the dataset is transcriptome-based, the authors should be cautious when interpreting non-detection of transcripts as evidence of gene loss. Transcript recovery can be influenced by expression levels, culture conditions, assembly completeness, and other technical factors. A brief discussion of this limitation would strengthen the manuscript.

**>>>>AU: We have added the statement “confirming this absence will require broader taxonomic sampling, supported by contiguous genome assemblies rather than relying largely on transcriptomic data.” and toned down the statement on PYL loss-necessitated rewiring.**

7. Line 543. LG+I+G4 is not a simplified mixture model. In IQ-TREE, it is classified as a standard empirical amino acid substitution model with site-rate heterogeneity.
**>>>>AU: We fully agree. Corrected.**

8. Figure 3. Both the terms Zygnematophyte and Zygnematophyceae are used in this figure. For consistency, I suggest using the same terminology throughout all panels. In addition, the abbreviation "PLT" presumably refers to land plants, but this is not defined in the figure or legend.

**>>>>AU: We have harmonized this.**

9. Title. The title is somewhat unusual in its wording. The authors may wish to consider a more direct formulation that would be readily accessible to a broader readership.

**>>>>AU: We fully agree. Yet, we have long thought about how to best highlight the finding that was the biggest surprise and has in our view also the highest “newsworthiness”.**

10. Line 41 of the Summary. The sentence ending with "revealing that this algal class is ancient" could be made more precise by emphasizing the extent of genetic divergence rather than age per se.

**>>>>AU: Good point. We have removed this and let the following sentences speak for themselves (because these important points are outlined there, both the genetic divergence and the radiation).**

11. Language and style. The manuscript contains a small number of grammatical and stylistic issues that should be addressed during revision. For example:
-Line 98: replace "pronounced ancient divergence" with "pronounced genetic divergence".

**>>>>AU: Changed accordingly.**

-Line 136: replace "6.2 longer" with "6.2-fold longer".

**>>>>AU: Changed accordingly.**

-Lines 139-141: revise the sentence for improved readability.

**>>>>AU: We have revised this section.**

__________________________________________________
In compliance with data protection regulations, you may request that we remove your personal registration details at any time.  (Use the following URL: <https://www.editorialmanager.com/pbiology/login.asp?a=r>). Please contact the publication office if you have any questions.
