# Supplementary material for: A deep genetic structure phylogenomically frames the closest algal relatives of land plants
Source: PLoS Biol. 2026 Nov 3;24(11):e3004005. doi: 10.1371/journal.pbio.3004005 (PMC13730384; doi:10.1371/journal.pbio.3004005)
Supplement: Peer review history

# Editor report: Decision Letter 0

**DOI:** 10.1371/journal.pbio.3004005.r001

**Authors:** Ankiit Ahluwalia

30 Jun 2026

Dear Dr de Vries,

Thank you for submitting your manuscript entitled "A deep genetic structure phylogenomically frames the closest algal relatives of land plants" for consideration as a Short Reports by PLOS Biology.

My apologies for late response as I was travelling and attending conferences. Your manuscript has now been evaluated by the PLOS Biology editorial staff and I am writing to let you know that we would like to send your submission out for external peer review.

However, before we can send your manuscript to reviewers, we need you to complete your submission by providing the metadata that is required for full assessment. To this end, please login to Editorial Manager where you will find the paper in the 'Submissions Needing Revisions' folder on your homepage. Please click 'Revise Submission' from the Action Links and complete all additional questions in the submission questionnaire.

Once your full submission is complete, your paper will undergo a series of checks in preparation for peer review. After your manuscript has passed the checks it will be sent out for review. To provide the metadata for your submission, please Login to Editorial Manager (https://www.editorialmanager.com/pbiology) within two working days, i.e. by Jul 02 2026 11:59PM.

If your manuscript has been previously peer-reviewed at another journal, PLOS Biology is willing to work with those reviews in order to avoid re-starting the process. Submission of the previous reviews is entirely optional and our ability to use them effectively will depend on the willingness of the previous journal to confirm the content of the reports and share the reviewer identities. Please note that we reserve the right to invite additional reviewers if we consider that additional/independent reviewers are needed, although we aim to avoid this as far as possible. In our experience, working with previous reviews does save time.

If you would like us to consider previous reviewer reports, please edit your cover letter to let us know and include the name of the journal where the work was previously considered and the manuscript ID it was given. In addition, please upload a response to the reviews as a 'Prior Peer Review' file type, which should include the reports in full and a point-by-point reply detailing how you have or plan to address the reviewers' concerns.

During the process of completing your manuscript submission, you will be invited to opt-in to posting your pre-review manuscript as a bioRxiv preprint. Visit http://journals.plos.org/plosbiology/s/preprints for full details. If you consent to posting your current manuscript as a preprint, please upload a single Preprint PDF.

Feel free to email us at plosbiology@plos.org if you have any queries relating to your submission.

Kind regards,

Ankiit

Ankiit Ahluwalia,

PLOS Biology

aahluwalia@plos.org

# Peer review: Decision Letter 1

**DOI:** 10.1371/journal.pbio.3004005.r002

**Authors:** Ankiit Ahluwalia

10 Aug 2026

Dear Dr de Vries,

Thank you for your patience while your manuscript "A deep genetic structure phylogenomically frames the closest algal relatives of land plants" was peer-reviewed at PLOS Biology. It has now been evaluated by the PLOS Biology editors, an Academic Editor with relevant expertise, and by several independent reviewers.

Based on the reviews and on our Academic Editor's assessment of your revision, we are likely to accept this manuscript for publication, provided you satisfactorily address the remaining points raised by the reviewers. Please also make sure to address the following data and other policy-related requests.

IMPORTANT - please attend to the following:

a) Thank you for providing the underlying data and code on GitHub. However, because GitHub repositories can be readily changed or deleted, please make a permanent, DOI assigned copy (e.g. in Zenodo) and provide the corresponding URL.

b) Please make the data deposited on Zenodo (https://doi.org/10.5281/zenodo.18392035) publicly available at this stage. For instance, we require the data directly underlying the trees shown in Figs 1A, 2, 3ABC and S1AB (the data required to generate these trees - potentially just a treefile).

c) Please clearly cite the location of the data in all relevant main and supplementary figure legends, for example: “The data underlying this figure can be found at doi.org/10.xxx/zenodo.xxxxx.” We understand that this may appear repetitive, but it helps make the figures and their legends more self contained.

d) Please make the raw data deposited on NCBI Bioproject (PRJNA1416270) publicly available at this stage.

e) Please include the URLs of your funders in the Financial Disclosure statement.

f) Reviewer3 in their point 9 suggests improving the title. I am happy to discuss alternatives by email.

g) Our Academic editor has some comments (below). Please address these presentational comments

"One minor point is that I find the three main figures to be very geared toward an algal/plant audience. It would be nice if the authors could edit these to be a bit more accessible to a general audience, e.g. 1) making the images of the organisms more directly linked to the specific phylogenetic clades (I am unclear currently which lineages match to which photos), 2) highlight land plants vs. other algae in Figure 2 so the cellular transitions make a bit more sense, and 3) Perhaps add some further annotation or context for Figure 3 so it doesn't come across as so genetics-heavy. I think improving the figures with these minor edits would be better for the broad readership of PLOS Biology."

As you address these items, please take this last chance to review your reference list to ensure that it is complete and correct. If you have cited papers that have been retracted, please include the rationale for doing so in the manuscript text, or remove these references and replace them with relevant current references. Any changes to the reference list should be mentioned in the cover letter that accompanies your revised manuscript.

In addition to these revisions, you may need to complete some formatting changes, which you will receive in a follow up email. A member of our team will be in touch with a set of requests shortly. If you do not receive a separate email within a few days, please assume that checks have been completed, and no additional changes are required.

We expect to receive your revised manuscript within two weeks.

To submit your revision, please go to https://www.editorialmanager.com/pbiology/ and log in as an Author. Click the link labelled 'Submissions Needing Revision' to find your submission record. Your revised submission must include the following:

- a cover letter that should detail your responses to any editorial requests, if applicable, and whether changes have been made to the reference list

- a Response to Reviewers file that provides a detailed response to the reviewers' comments (if applicable, if not applicable please do not delete your existing 'Response to Reviewers' file.)

- a track-changes file indicating any changes that you have made to the manuscript.

NOTE: If Supporting Information files are included with your article, note that these are not copyedited and will be published as they are submitted. Please ensure that these files are legible and of high quality (at least 300 dpi) in an easily accessible file format. For this reason, please be aware that any references listed in an SI file will not be indexed. For more information, see our Supporting Information guidelines:

https://journals.plos.org/plosbiology/s/supporting-information

*Published Peer Review History*

Please note that you may have the opportunity to make the peer review history publicly available. The record will include editor decision letters (with reviews) and your responses to reviewer comments. If eligible, we will contact you to opt in or out. Please see here for more details:

https://plos.org/published-peer-review-history/

*Press*

Should you, your institution's press office or the journal office choose to press release your paper, please ensure you have opted out of Early Article Posting on the submission form. We ask that you notify us as soon as possible if you or your institution is planning to press release the article.

*Protocols deposition*

To enhance the reproducibility of your results, we recommend that if applicable you deposit your laboratory protocols in protocols.io, where a protocol can be assigned its own identifier (DOI) such that it can be cited independently in the future. Additionally, PLOS ONE offers an option for publishing peer-reviewed Lab Protocol articles, which describe protocols hosted on protocols.io. Read more information on sharing protocols at https://plos.org/protocols?utm_medium=editorial-email&utm_source=authorletters&utm_campaign=protocols

Please do not hesitate to contact me should you have any questions.

Sincerely,

Ankiit

Ankiit Ahluwalia,

aahluwalia@plos.org

PLOS Biology

------------------------------------------------------------------------

****[DELETE AS APPROPRIATE]****

ETHICS STATEMENT:

-- Please include the full name of the IACUC/ethics committee that reviewed and approved the animal care and use protocol/permit/project license. Please also include an approval number.

-- Please include the specific national or international regulations/guidelines to which your animal care and use protocol adhered. Please note that institutional or accreditation organization guidelines (such as AAALAC) do not meet this requirement.

-- Please include information about the form of consent (written/oral) given for research involving human participants. All research involving human participants must have been approved by the authors' Institutional Review Board (IRB) or an equivalent committee, and must have been conducted according to the principles expressed in the Declaration of Helsinki.

------------------------------------------------------------------------

DATA POLICY:

You may be aware of the PLOS Data Policy, which requires that all data be made available without restriction: http://journals.plos.org/plosbiology/s/data-availability. For more information, please also see this editorial: http://dx.doi.org/10.1371/journal.pbio.1001797

Note that we do not require all raw data. Rather, we ask that all individual quantitative observations that underlie the data summarized in the figures and results of your paper be made available in one of the following forms:

1) Supplementary files (e.g., excel). Please ensure that all data files are uploaded as 'Supporting Information' and are invariably referred to (in the manuscript, figure legends, and the Description field when uploading your files) using the following format verbatim: S1 Data, S2 Data, etc. Multiple panels of a single or even several figures can be included as multiple sheets in one excel file that is saved using exactly the following convention: S1_Data.xlsx (using an underscore).

2) Deposition in a publicly available repository. Please also provide the accession code or a reviewer link so that we may view your data before publication.

Regardless of the method selected, please ensure that you provide the individual numerical values that underlie the summary data displayed in the following figure panels as they are essential for readers to assess your analysis and to reproduce it:

[Figs….]

NOTE: the numerical data provided should include all replicates AND the way in which the plotted mean and errors were derived (it should not present only the mean/average values).

Please also ensure that figure legends in your manuscript include information on where the underlying data can be found, and ensure your supplemental data file/s has a legend.

Please ensure that your Data Statement in the submission system accurately describes where your data can be found.

------------------------------------------------------------------------

APPROPRIATE DATA PRESENTATION?

(If you think the paper is not using best practice, add the following)

- Please ensure that you are using best practice for statistical reporting and data presentation. These are our guidelines https://journals.plos.org/plosbiology/s/best-practices-in-research-reporting#loc-statistical-reporting and a useful resource on data presentation https://journals.plos.org/plosbiology/article?id=10.1371/journal.pbio.1002128

- If you are reporting experiments where n ≤ 5, please plot each individual data point.

------------------------------------------------------------------------

CODE POLICY

Per journal policy, if you have generated any custom code during the course of this investigation, please make it available without restrictions. Please ensure that the code is sufficiently well documented and reusable, and that your Data Statement in the Editorial Manager submission system accurately describes where your code can be found. More information on our Code Policy, what and how to share can be found here: https://journals.plos.org/plosbiology/s/code-availability

Please note that we cannot accept sole deposition of code in GitHub, as this could be changed after publication. However, you can archive this version of your publicly available GitHub code to Zenodo. Once you do this, it will generate a DOI number, which you will need to provide in the Data Accessibility Statement (you are welcome to also provide the GitHub access information). See the process for doing this here: https://docs.github.com/en/repositories/archiving-a-github-repository/referencing-and-citing-content

------------------------------------------------------------------------

SPECIES INDICATED IN THE ABSTRACT?

- Please note that per journal policy, the model system/species studied should be clearly stated in the abstract of your manuscript.

**EDITOR, CONSIDER ALSO INCLUDING ORGANISM IN THE TITLE, IF SENSICAL/RELEVANT AND/OR TO AVOID BEING MISLEADING/OVERREACHING (not always preferred)**

------------------------------------------------------------------------

BLOT AND GEL REPORTING REQUIREMENTS:

We require the original, uncropped and minimally adjusted images supporting all blot and gel results reported in an article's figures or Supporting Information files. We will require these files before a manuscript can be accepted so please prepare and upload them now. Please carefully read our guidelines for how to prepare and upload this data: https://journals.plos.org/plosbiology/s/figures#loc-blot-and-gel-reporting-requirements

------------------------------------------------------------------------

DATA NOT SHOWN?

- Please note that per journal policy, we do not allow the mention of "data not shown", "personal communication", "manuscript in preparation" or other references to data that is not publicly available or contained within this manuscript. Please either remove mention of these data or provide figures presenting the results and the data underlying the figure(s).

------------------------------------------------------------------------

Reviewer remarks:

Reviewer #1: A deep genetic structure phylogenomically frames the closest algal relatives of land Plants

Bierenbroodspot et al

This manuscript presents a comprehensive phylogenomic analysis of streptophyte algae to clarify the evolutionary origin of land plants. Using a genome-scale dataset, the authors resolve the deep evolutionary relationships within streptophyte algae and provide new views on the relationships among the closest algal relatives of embryophytes. I think the study significantly advances our understanding of the evolutionary transition from aquatic algae to terrestrial plants and provides a framework for investigating the origin of key land plant traits.

Overall, I consider this to be a high-quality study with the potential for substantial impact. It is likely to become an important reference for research on early streptophyte evolution and the evolutionary context of land plant origins. I found the analyses rigorous, and I do not have any major concerns regarding the study design. The remaining uncertainties are largely those inherent to deep phylogenomic reconstruction rather than shortcomings of the work itself.

I have a few major comments:

1) Ancestral reconstruction of morphological traits: one potential caveat concerns the reconstruction of morphological characters. I believe many streptophyte algae have complex life cycles, with different generations or life stages exhibiting distinct morphologies. If this is the case for some of the taxa included here, trait coding based on a single life stage could bias ancestral state reconstruction. I appreciate that complete life cycle information may not be available for many species, but I think this limitation should be explicitly acknowledged and discussed.

2) Definition of genomic streamlining: it is not entirely clear how the trait "genomic streamlining" was defined and quantified. I recommend providing additional methodological details describing the criteria used to assign this trait.

3) Interpretation of PYL loss:the discussion of PYL loss should be interpreted more cautiously. Demonstrating true gene loss can be challenging, particularly when genome assemblies are incomplete or when transcriptome-based data are used, where the absence of expression does not necessarily imply gene absence. It would be helpful if the authors discussed these limitations and moderated the statement that PYL loss "underpins the idea that extensive rewiring in the homologous chassis occurred."

4) Description of morphological traits: since ancestral reconstruction of morphological traits is a central component of the study, I think the manuscript would benefit from providing clearer definitions of the characters used. For example, I was not entirely sure what was meant by a "chain-like filament," and I felt I should not have to consult reference 97 to understand such an important character. Similarly, terms such as "desmid cell" may not be familiar to a broad readership. Brief definitions or illustrations within the manuscript would make the work more accessible.

5) Evolution of multicellularity and body plans. The manuscript discusses the independent evolution of filamentous growth several times, but I found it equally interesting that the "macrophyte" body plan also appears to have evolved independently on multiple occasions. Moreover, unless I have misunderstood the reconstruction, these transitions seem to occur directly from unicellular ancestors rather than through filamentous intermediates. If this interpretation is correct, I think it deserves further discussion, as it has potentially interesting implications for the evolution of complex multicellularity in streptophyte algae.

6) Evolutionary models for trait transitions. I also wondered whether the authors considered using phyllogenetic comparative methods to estimate transition probabilities among traits. Such approaches might provide complementary insights into the evolutionary dynamics of these characters. If the authors considered these analyses but chose not to pursue them, it would be useful to briefly explain why.

Minor

Please add scale bar to photos in Figure 1

Reviewer #2: In this study, Bierenbroodspot and coauthors provide 43 novel transcriptomes and phylogenomic analyses within the class Zygnematophyceae.

This class of algae is the closest streptophyte algal relative of embryophytes.

Beyond the detailed phylogeny of this class, this study also addresses more general issues such as the evolution of multicellularity and the evolution of gene families associated with the ecological success of land plants.

The authors completed the available sequence data with 43 transcriptomes from approximately 36 species, adding up to 104 zygnematophycean algae, nine other streptophyte algae, eight land plants, and four chlorophyte algae as an outgroup.

The authors first confirm the phylogenetic position of the Zygnematophyceae class as the closest streptophyte algal relatives of embryophytes (Figure 1).

They use ancestral state reconstruction to infer the ancestral morphology (unicellular/ filament/ sarcinoid) of this class. The analyses suggest the ancestral state is unicellular and that there have been seven independent transitions to a filamentous body plan (Figure 2).

They then investigate a few families : the abscisic acid receptor gene, and gene families from the phenylpropanoid pathway (Figure 3).

The manuscript is very well written, synthetic, the analyses and results are sound, and I have only minor comments : I think providing additional context and information would further increase its readability. Below are some suggestions in this direction.

1.As a non-Zygnematophyceae expert, I believe this will be the case for many readers, I would have appreciated some more general context about previous quests of the gene families involved in terrestralisation (e.g. https://doi.org/10.1371/journal.pbio.3002608), as well as the ancestry of the genes and gene families the authors target in this study. Could you provide more information about the presence/absence of homologs in more ancestral lineages, before describing the independent gene family expansions in Zygnematophyceae and Embryophytes ? May the annotation of the black branches in Figure 3 provide this information ?

2.Methods section : line 141 Could you clarify why you excluded clades when only one protein contained the domain, Couldn't it be that there is juste one copy of this gene in the clade ?

Reviewer #3: This manuscript presents a major phylogenomic investigation of the Zygnematophyceae, the closest algal relatives of land plants. By generating 43 new transcriptomes and integrating them with publicly available datasets, the authors establish an extensive phylogenomic framework comprising 104 zygnematophyte taxa and 2,243 loci. The resulting dataset considerably expands taxonomic representation across the group and provides an important resource for future studies of streptophyte algal evolution and plant terrestrialization.

Overall, I found the manuscript to be well written, the analytical framework carefully designed, and the conclusions largely supported by the data. In particular, the breadth of taxon sampling represents a significant advance over previous studies and allows the authors to address several evolutionary questions within a much stronger phylogenetic context than has previously been possible. The paper will be of broad interest to researchers working on plant evolution, algal diversity, and the origins of land plant traits.

I have only a few suggestions that I believe would further strengthen the manuscript.

COMMENTS

1. Figure 1. I recommend indicating in panel A which taxa were newly sampled in this study. In addition, some labels and clade names could be enlarged to improve readability.

2. Lines 136-142. The discussion of the exceptionally long branch leading to extant Spirogyrales is thought-provoking. However, some of the interpretations presented, such as extensive extinction or the existence of large amounts of unsampled diversity, are necessarily speculative. I suggest presenting these possibilities more explicitly as alternative hypotheses rather than preferred explanations. Additional explanations for long branches include accelerated rates of molecular evolution, heterogeneous selection pressures, compositional biases, and transcriptome-derived biases.

3. Lines 92-96 and 515-518. The ancestral character state reconstruction constitutes an important aspect of the manuscript, particularly the inference of repeated origins of filamentous growth and a unicellular ancestor of Zygnematophyceae. It would be helpful if the authors could provide additional methodological details, as the description in the Methods section is currently quite brief and may make the analysis difficult to reproduce. More detailed information may be available on the authors' Zenodo and GitHub sites, but I was unable to access them. A brief discussion of the assumptions underlying the reconstruction model would also be useful.

4. Figure 2 and legend. Additional details regarding the output of the ancestral character state reconstruction would help readers interpret the results. In particular, how should circles containing multiple-colored sectors be interpreted? For example, what is the inferred likelihood of different body plans for the common ancestor of Zygnematophyceae and land plants? Was this ancestor more likely to have been unicellular than multicellular? Uncertainty in reconstructed states should be shown if possible.

5. Lines 209-211. The authors state that "both in the case of the Charophyceae and the discoidal Coleochaete species, the complexity of body plans is likely the result of secondary gains." This raises the question of whether the common ancestor of Charophyceae and Coleochaetophyceae was also unicellular, as inferred for the common ancestor of Zygnematophyceae. Additional discussion of this point would be helpful.

6. Lines 229-232. The analyses of PYL homolog distribution across Zygnematophyceae are interesting and provide valuable insights into the evolutionary history of this gene family. However, because much of the dataset is transcriptome-based, the authors should be cautious when interpreting non-detection of transcripts as evidence of gene loss. Transcript recovery can be influenced by expression levels, culture conditions, assembly completeness, and other technical factors. A brief discussion of this limitation would strengthen the manuscript.

7. Line 543. LG+I+G4 is not a simplified mixture model. In IQ-TREE, it is classified as a standard empirical amino acid substitution model with site-rate heterogeneity.

8. Figure 3. Both the terms Zygnematophyte and Zygnematophyceae are used in this figure. For consistency, I suggest using the same terminology throughout all panels. In addition, the abbreviation "PLT" presumably refers to land plants, but this is not defined in the figure or legend.

9. Title. The title is somewhat unusual in its wording. The authors may wish to consider a more direct formulation that would be readily accessible to a broader readership.

10. Line 41 of the Summary. The sentence ending with "revealing that this algal class is ancient" could be made more precise by emphasizing the extent of genetic divergence rather than age per se.

11. Language and style. The manuscript contains a small number of grammatical and stylistic issues that should be addressed during revision. For example:

-Line 98: replace "pronounced ancient divergence" with "pronounced genetic divergence".

-Line 136: replace "6.2 longer" with "6.2-fold longer".

-Lines 139-141: revise the sentence for improved readability.

# Author comment: Author response to Decision Letter 2

**DOI:** 10.1371/journal.pbio.3004005.r003

1 Sep 2026

Attachment Submitted filename: ResponseLetter.docx

# Editor report: Decision Letter 2

**DOI:** 10.1371/journal.pbio.3004005.r004

**Authors:** Ankiit Ahluwalia

3 Sep 2026

Dear Dr de Vries,

Thank you for the submission of your revised Short Report "A deep genetic structure phylogenomically frames the closest algal relatives of land plants" for publication in PLOS Biology. I'm handling your paper temporarily while my colleague Dr Ankiit Ahluwalia is out of the office. On behalf of my colleagues and the Academic Editor, Holly Bik, I am pleased to say that we can in principle accept your manuscript for publication, provided you address any remaining formatting and reporting issues. These will be detailed in an email you should receive within 2-3 business days from our colleagues in the journal operations team; no action is required from you until then. Please note that we will not be able to formally accept your manuscript and schedule it for publication until you have completed any requested changes.

IMPORTANT: I've asked my colleagues to include the following request alongside their own: Many thanks for providing the data and code. To make the Figs more standalone, please cite the location of the data clearly in all relevant main and supplementary Figure legends, e.g. “The data and code needed to generate this Figure can be found in https://zenodo.org/records/XXXXXXXX"

Please take a minute to log into Editorial Manager at http://www.editorialmanager.com/pbiology/, click the "Update My Information" link at the top of the page, and update your user information to ensure an efficient production process.

PRESS: We frequently collaborate with press offices. If your institution or institutions have a press office, please notify them about your upcoming paper at this point, to enable them to help maximise its impact. If the press office is planning to promote your findings, we would be grateful if they could coordinate with biologypress@plos.org. If you have previously opted in to the early version process, we ask that you notify us immediately of any press plans so that we may opt out on your behalf.

We also ask that you take this opportunity to read our Embargo Policy regarding the discussion, promotion and media coverage of work that is yet to be published by PLOS. As your manuscript is not yet published, it is bound by the conditions of our Embargo Policy. Please be aware that this policy is in place both to ensure that any press coverage of your article is fully substantiated and to provide a direct link between such coverage and the published work. For full details of our Embargo Policy, please visit http://www.plos.org/about/media-inquiries/embargo-policy/.

Thank you again for choosing PLOS Biology for publication and supporting Open Access publishing. We look forward to publishing your study.

Sincerely,

Roli Roberts

Roland G Roberts PhD

Senior Editor

PLOS Biology

rroberts@plos.org

on behalf of

Ankiit Ahluwalia

Associate Editor

PLOS Biology

aahluwalia@plos.org
